# Aspirin blocks formation of metastatic intravascular niches by inhibiting platelet-derived COX-1/thromboxane A_2_

**DOI:** 10.1172/JCI121985

**Published:** 2019-03-25

**Authors:** Serena Lucotti, Camilla Cerutti, Magali Soyer, Ana M. Gil-Bernabé, Ana L. Gomes, Philip D. Allen, Sean Smart, Bostjan Markelc, Karla Watson, Paul C. Armstrong, Jane A. Mitchell, Timothy D. Warner, Anne J. Ridley, Ruth J. Muschel

**Affiliations:** 1 Cancer Research UK and MRC Oxford Institute for Radiation Oncology, Department of Oncology, University of Oxford, Oxford, United Kingdom.; 2 Randall Division of Cell and Molecular Biophysics, King’s College London, New Hunt’s House, Guy’s Campus, London, United Kingdom.; 3 Centre for Immunobiology, Blizard Institute, Barts and The London School of Medicine and Dentistry, Queen Mary University of London, London, United Kingdom.; 4 Cardiothoracic Pharmacology, Vascular Biology, National Heart and Lung Institute, Imperial College London, London, United Kingdom.

**Keywords:** Oncology, Therapeutics, Cancer, Platelets, Thrombosis

## Abstract

Because metastasis is associated with the majority of cancer-related deaths, its prevention is a clinical aspiration. Prostanoids are a large family of bioactive lipids derived from the activity of cyclooxygenase-1 (COX-1) and COX-2. Aspirin impairs the biosynthesis of all prostanoids through the irreversible inhibition of both COX isoforms. Long-term administration of aspirin leads to reduced distant metastases in murine models and clinical trials, but the COX isoform, downstream prostanoid, and cell compartment responsible for this effect are yet to be determined. Here, we have shown that aspirin dramatically reduced lung metastasis through inhibition of COX-1 while the cancer cells remained intravascular and that inhibition of platelet COX-1 alone was sufficient to impair metastasis. Thromboxane A_2_ (TXA_2_) was the prostanoid product of COX-1 responsible for this antimetastatic effect. Inhibition of the COX-1/TXA_2_ pathway in platelets decreased aggregation of platelets on tumor cells, endothelial activation, tumor cell adhesion to the endothelium, and recruitment of metastasis-promoting monocytes/macrophages, and diminished the formation of a premetastatic niche. Thus, platelet-derived TXA_2_ orchestrates the generation of a favorable intravascular metastatic niche that promotes tumor cell seeding and identifies COX-1/TXA_2_ signaling as a target for the prevention of metastasis.

## Introduction

Prostanoids are a family of bioactive lipids comprising prostaglandins (e.g., PGD_2_, PGE_2_, PGF_2a_), thromboxane A_2_ (TXA_2_), and prostacyclin (PGI_2_). The rate-limiting step of prostanoid biosynthesis is catalyzed by cyclooxygenase (COX), an enzyme with 2 isoforms, COX-1 and COX-2. Both COX-1 and COX-2 have virtually identical enzymatic activity, mediating the conversion of arachidonic acid into PGG_2_ and then into PGH_2_, the common precursor of all prostanoids (1). However, the spectrum of prostanoids synthesized by each isoform differs in vivo as a result of distinct expression patterns and functional coupling to prostanoid synthases in different cell types (2). For example, COX-2 is induced in endothelial cells and macrophages during inflammation and wound healing and couples with PGE_2_ synthase in those cells to produce proinflammatory PGE_2_ (2, 3). In contrast, COX-1 is constitutively expressed. In platelets COX-1 couples with TXA_2_ synthase (TXAS) to generate prothrombotic TXA_2_ upon procoagulant stimuli (e.g., collagen, thrombin, and adenosine diphosphate [ADP]) (4–6). Because of the differential expression of prostanoid synthases and COX-1 and COX-2, the activity of the 2 isoforms is rarely redundant.

The importance of COX and prostanoid pathways in metastasis is apparent from reports showing that their inhibition greatly curtails metastasis. NSAIDs, including aspirin, that inhibit both COX-1 and COX-2 generally reduce metastasis in clinical studies and murine models (7–9). In some reports specific COX-2 inhibition blocks metastasis (10, 11), but not in others (12). Looking at the downstream prostanoids, inhibition of TXA_2_ or of PGE_2_ synthesis also reduces metastasis in animal models, while PGI_2_ has been reported to inhibit metastasis (11–18), with some exceptions (12, 19). These reports raise the question of whether some prostanoids might be suitable targets for metastasis prevention or therapy.

The possibility of using COX or prostanoid synthesis inhibition as a preventive strategy for metastasis has been highlighted by both clinical and experimental studies. Aspirin is given clinically in a variety of doses to reduce cardiovascular events or inflammation. Because of its unique combination of irreversible inhibition of COX enzymes and short circulating half-life, low-dose aspirin preferentially inhibits COX-1 in platelets, reducing the production of prothrombotic TXA_2_ and other prostanoids (20). Thus, low-dose aspirin is given for prophylaxis of myocardial infarction and stroke. Higher doses of aspirin inhibit both COX isoforms in other tissues (21). In particular, the reduction of COX-2–derived PGE_2_ exerts antiinflammatory effects. Case-control studies and meta-analysis of randomized controlled trials have shown that aspirin given for these unrelated purposes reduces metastatic cancer (22, 23). This effect was significant over a range of primary tumor types, with a more prominent effect on adenocarcinomas (23). While aspirin also reduces metastasis in murine models (8, 11, 24, 25), it is not generally established whether the reduction of metastasis can be attributed to inhibition of COX-1 or COX-2.

During hematogenous metastasis, tumor cells have adapted to co-opt diverse cell types, including platelets, endothelial cells, and immune cells, to survive the hostile environment of the blood circulation (26). In particular, cancer cells engage platelets to form small aggregates or clots on their surface through expression of tissue factor (TF) (27) or P-selectin ligands (28, 29). Platelet aggregation and activation itself supports tumor cell survival in the circulation, either directly or through recruitment of myeloid cells, and can initiate cancer cell spreading on the surface of the blood vessel and transendothelial migration (27, 29–33). Different prostanoids are synthetized by a variety of cells in the vascular niche and are essential to maintain cardiovascular homeostasis, but the levels and source of prostanoids often change dramatically between physiological and pathological conditions (3, 34, 35). Thus, in addition to uncertainly as to which COX isoform is responsible for supporting metastasis, it is unclear in which cell compartment they are crucial and which prostanoids might be responsible for their effects.

Here we demonstrate using a variety of different models that specific inhibition of COX-1 in platelets is sufficient to inhibit metastasis to the same extent as aspirin whereas inhibition of COX-2 does not reduce metastatic colonization. We further show that COX-1 blockade leading to inhibition of TXA_2_ synthesis in platelets is sufficient to inhibit metastasis. Lastly we provide evidence that the antimetastatic effect of COX-1 inhibition is generally limited to the early stages of metastasis and that inhibition of COX-1 or of TXA_2_ synthesis prevents the formation of an intravascular metastatic and premetastatic niche.

## Results

### Reduction of metastasis by aspirin correlates with the inhibition of thrombosis.

**Figure 1:**
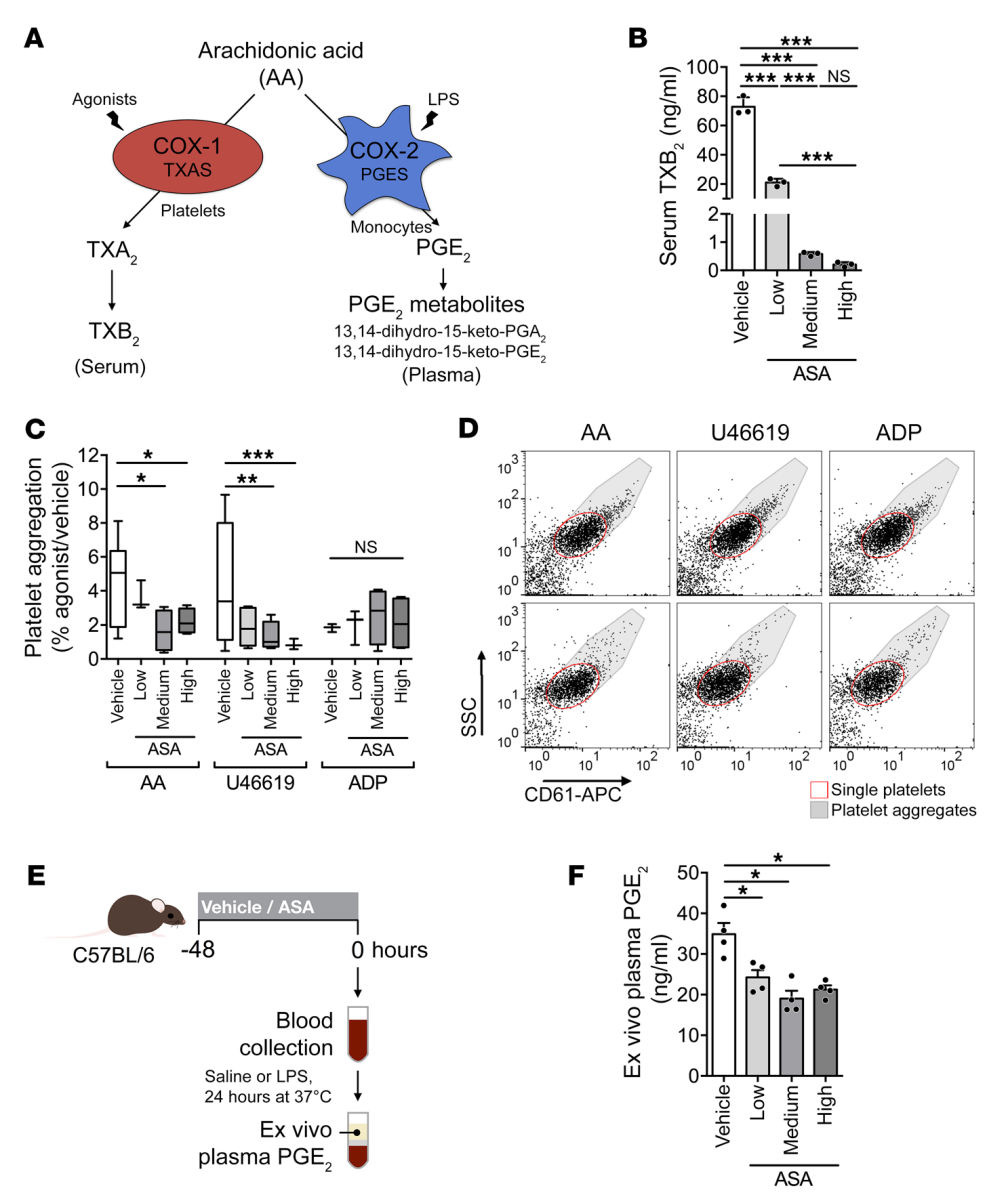
Medium-dose aspirin reduces COX-1–dependent platelet aggregation. (**A**) Diagram of COX-1 and COX-2 products. Serum TXB_2_ represents COX-1 activity in platelets. Plasma PGE_2_ represents COX-2 activity in monocytes. (**B**) TXB_2_ in serum from C57BL/6 mice treated with vehicle or aspirin (ASA; low, medium, or high) for 2 days before blood sampling (*n* = 3). (**C** and **D**) Agonist-induced aggregation of CD61-stained platelets from mice treated with vehicle or aspirin for 2 days. Arachidonic acid, U46619, and ADP were the agonists (*n* = 7 for vehicle group, 4 for all other groups). (**E** and **F**) Experimental design (**E**) and ex vivo PGE_2_ levels (**F**) in plasma from mice in **B** (*n* = 4). Data are represented as mean + SD (**B** and **F**), mean ± range (**C**). One-way ANOVA with Tukey’s multiple-comparisons test. *0.01 < *P* ≤ 0.05; **0.001 < *P* ≤ 0.01; ***P ≤ 0.001.

We treated mice with different doses of aspirin (ASA; low, medium, and high), which were based on the low, medium, and high doses used in humans according to a body surface area dose conversion method and on previous literature (8, 36–38). Inhibition of COX-1 was evaluated using serum levels of TXB_2_, a stable metabolite of TXA_2_ generated by platelet COX-1 activity during clotting (ex vivo) (Figure 1A and ref. 39). Greater than 95% reduction in TXB_2_ ex vivo is thought to indicate physiological inhibition of COX-1 (40). The medium and high doses, but not the low dose, of aspirin reduced TXB_2_ by more than 95% (Figure 1B) and, accordingly, reduced COX-1–dependent (arachidonic acid and U46619, a stable analog of TXA_2_) agonist-induced platelet aggregation (Figure 1, C and D). COX-1–independent (ADP) platelet aggregation was not affected (Figure 1, C and D). Importantly, low-dose aspirin did not reduce serum TXB_2_ more than 95% over 6 days after the treatment began, suggesting that the drug does not accumulate over time (Supplemental Figure 1; supplemental material available online with this article; https://doi.org/10.1172/JCI121985DS1).

Since COX-2 is not significantly expressed in blood cells in the absence of inflammation, we assayed COX-2 inhibition using plasma PGE_2_ after COX-2 induction by LPS (Figure 1, A and E, and ref. 41). All doses of aspirin reduced plasma PGE_2_ levels, demonstrating inhibition of COX-2 (Figure 1F). Systemic PGE_2_ metabolites (PGE_2_M) were also reduced (Supplemental Figure 2). The antiinflammatory effect of low-dose aspirin has been previously suggested (37, 42). Thus aspirin inhibited COX-2 at all doses but only inhibited COX-1 with physiological significance at the medium and high doses. Hence, the medium dose is the minimum dose to achieve antithrombotic effects in our model, similar to low-dose aspirin in humans.

**Figure 2:**
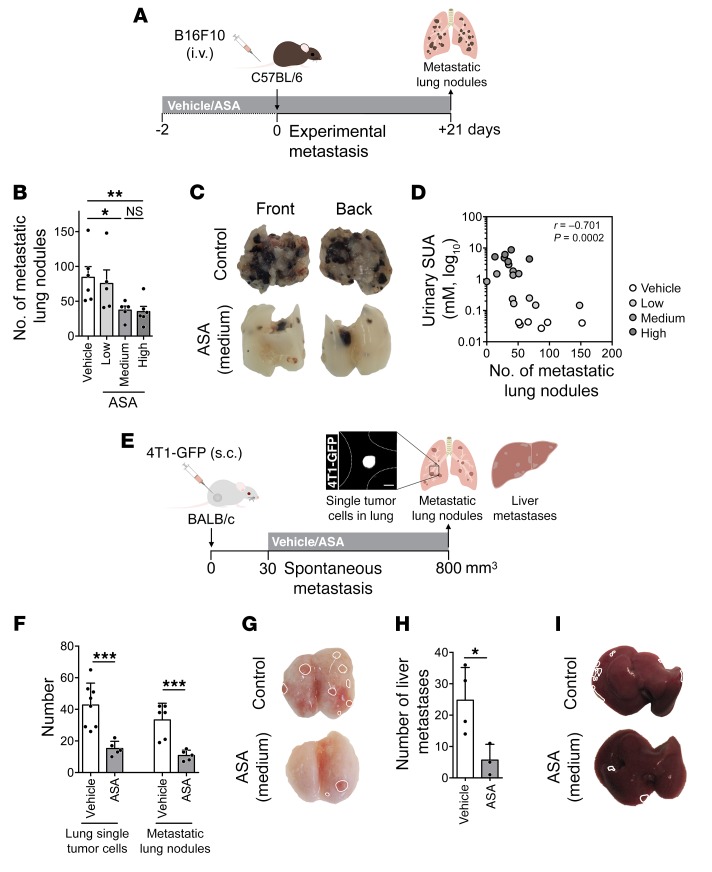
Aspirin reduces experimental metastasis. (**A**) Schematic representation of experimental metastasis assay. (**B** and **C**) B16F10 metastatic lung nodules in C57BL/6 mice treated with vehicle (*n* = 6) or aspirin (*n* = 5, 5, and 6). (**D**) Correlation plot of urinary concentration of salicyluric acid (SUA) versus the number of metastatic lung nodules of mice in **B**. (**E**) Schematic representation of spontaneous metastasis assay. Scale bar: 10 μm. (**F**–**I**) Single disseminated tumor cells in the lungs (**F**) and metastatic nodules to lungs (**F** and **G**) or liver (**H** and **I**) of BALB/c mice bearing 4T1-GFP tumors, treated with vehicle or aspirin (*n* = 8 and 5 in **F**, 4 and 3 in **H**). Data are represented as mean + SD. One-way ANOVA with Tukey’s multiple-comparisons test (**B**, **F**, and **H**); Spearman’s rank correlation (**D**). *0.01 < *P* ≤ 0.05; **0.001 < *P* ≤ 0.01; ***P ≤ 0.001.

The effects of aspirin on experimental metastasis were assessed in mice treated with aspirin starting 2 days before the i.v. injection of syngeneic B16F10 melanoma tumor cells (Figure 2A). Aspirin at the medium and high doses reduced the number of metastatic lung nodules by more than 50% (Figure 2, B and C). The number of colonies inversely correlated with aspirin intake (Figure 2D). Aspirin (medium dose) similarly reduced the number of metastatic lung nodules from MC-38-GFP, 4T1, and MDA-MB-231-CFP cells (Supplemental Figure 3), indicating a widespread inhibitory effect of aspirin on metastasis.

Spontaneous metastasis was also inhibited by aspirin. BALB/c mice with 4T1-GFP–derived subcutaneous tumors received vehicle or aspirin treatment (Figure 2E). Tumor growth was similar in both treatment groups, although aspirin treatment was associated with enhanced tumor regression (Supplemental Figure 4A). Aspirin decreased numbers of lung and liver metastases, of disseminated tumor cells in the lungs (Figure 2, F–I), and of circulating tumor cells (CTCs) (Supplemental Figure 4B) and the invasive ability of those CTCs (Supplemental Figure 4, C–E).

These data confirmed the inhibitory effect of aspirin on metastasis at doses that inhibit COX-1 activity and thrombosis, suggesting that aspirin affects metastasis establishment through an antithrombotic effect.

### COX-1 inhibition is sufficient to reduce metastasis.

**Figure 3:**
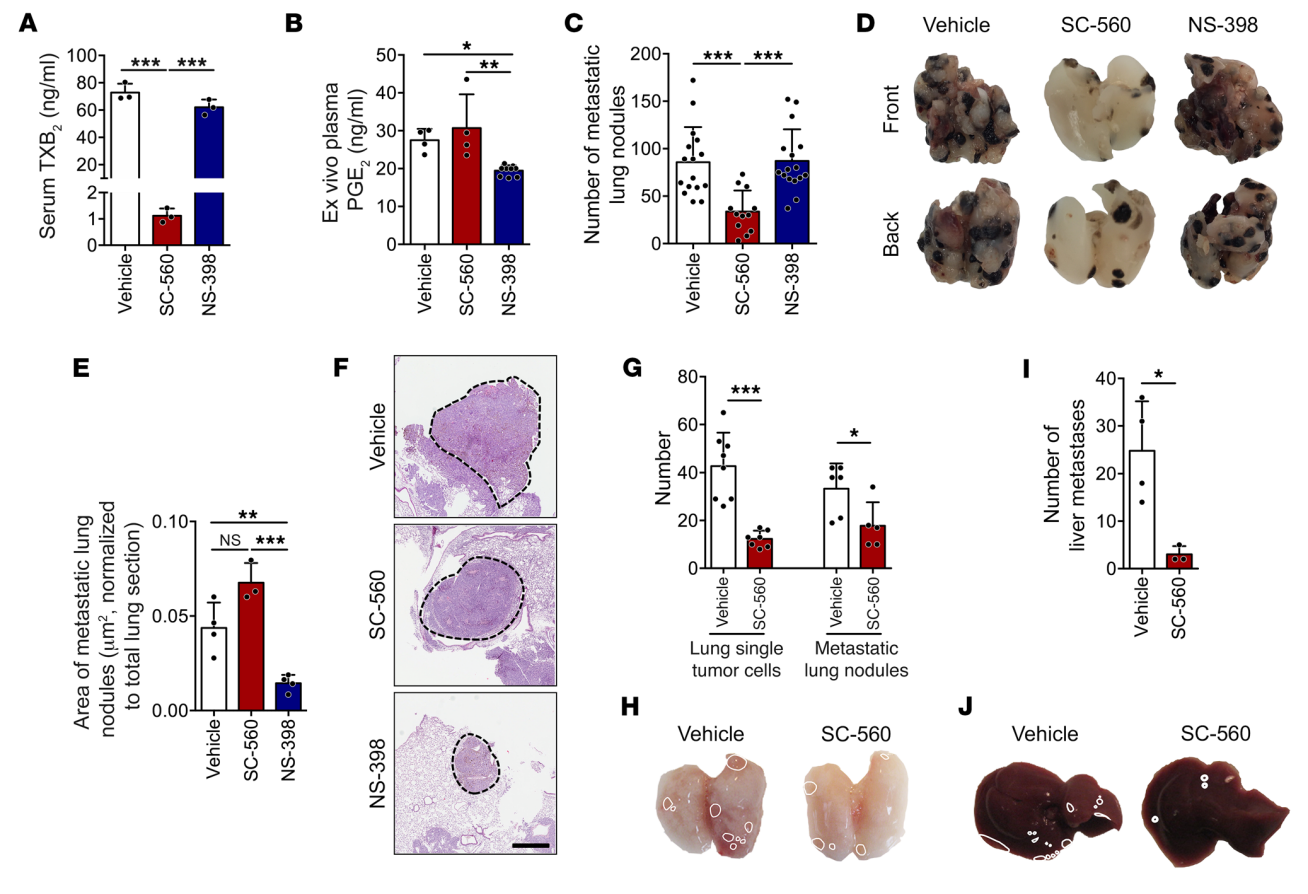
The inhibition of COX-1 is sufficient to reduce metastasis. (**A** and **B**) Concentration of TXB_2_ in serum (*n* = 3) (**A**) or PGE_2_ in LPS-stimulated plasma (*n* = 4) (**B**; see also Figure 1E) from mice treated with vehicle, SC-560, or NS-398 for 2 days. (**C** and **D**) B16F10 metastatic lung nodules in lungs from C57BL/6 mice treated with vehicle or drugs (*n* = 16, 12, and 16) as in Figure 2A. (**E** and **F**) Relative area (**E**) and representative images (**F**) of metastatic nodules from H&E-stained lung sections (**F**) (*n* = 4, 3, and 4; ≥3 nodules per lung). Scale bar: 700 μm. (**G**–**J**) Single disseminated tumor cells in lungs and metastatic lung nodules (**G** and **H**) and liver metastases (**I** and **J**) in BALB/c mice bearing 4T1-GFP tumors, treated with vehicle or SC-560 (*n* = 8 and 7), as in Figure 2E. Control groups are the same as in Figure 2, F and H. Data are represented as mean + SD. One-way ANOVA with Tukey’s multiple-comparisons test. *0.01 < *P* ≤ 0.05; **0.001 < *P* ≤ 0.01; ***P ≤ 0.001.

Since aspirin inhibits both COX-1 and COX-2 at metastasis-suppressive doses, we determined the effect on metastasis of selective inhibitors of COX-1 (SC-560) or COX-2 (NS-398). Isoform specificity was confirmed by reduction of serum TXB_2_ for COX-1 (Figure 3A) and plasma PGE_2_ for COX-2 (Figure 3B and Supplemental Figure 2). COX-1 inhibition by SC-560 significantly reduced the number of metastatic lung nodules from B16F10 cells (Figure 3, C and D) compared to the medium and high doses of aspirin (Figure 2B). COX-2 inhibition by NS-398 did not reduce the numbers (Figure 3, C and D), making it unlikely that metastatic seeding requires PGE_2_. However, NS-398–treated mice had smaller metastatic colonies (Figure 3, E and F), compatible with the reported involvement of COX-2 in tumor cell proliferation (15). Using other models, SC-560 also reduced experimental lung metastasis from MC-38-GFP, 4T1, and MDA-MB-231-CFP cells (Supplemental Figure 3) and spontaneous lung and liver metastasis, pulmonary dissemination (Figure 3, G–J), and CTCs and their invasiveness from 4T1-GFP tumor–bearing mice (Supplemental Figure 4, B–E).

**Figure 4:**
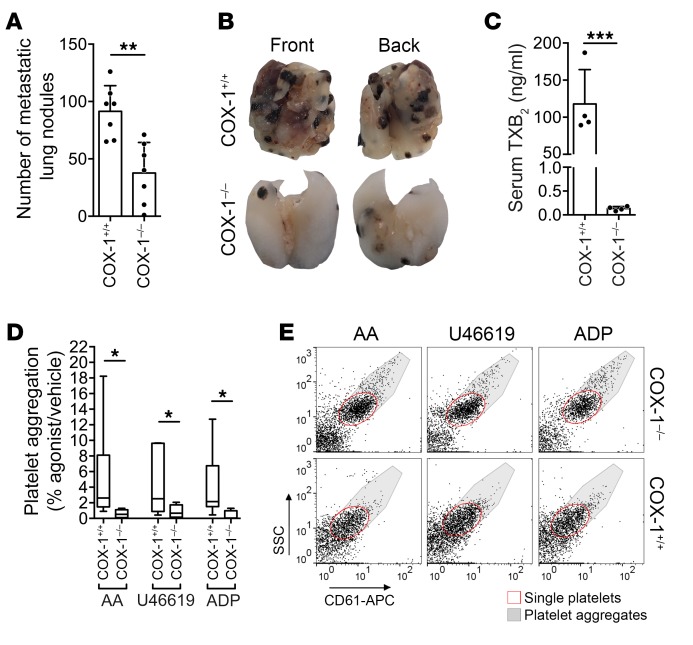
COX-1 deficiency decreases experimental lung metastasis. (**A** and **B**) B16F10 metastatic lung nodules in COX-1^+/+^ (C57BL/6) or COX-1^–/–^ mice (*n* = 7), as in Figure 2A. (**C**) Concentration of serum TXB_2_ from COX-1^+/+^ or COX-1^–/–^ mice (*n* = 4). (**D** and **E**) Agonist-induced aggregation of platelets from COX-1^+/+^ or COX-1^–/–^ mice (*n* = 8 and 4). Data are represented as mean + SD (**A** and **C**), median ± range (**D**). Unpaired *t* test, 2-tailed (**A** and **C**); 1-way ANOVA with Tukey’s multiple-comparisons test (**D**). *0.01 < *P* ≤ 0.05; **0.001 < *P* ≤ 0.01; ***P ≤ 0.001.

Fewer experimental metastases were generated by B16F10 cells in COX-1^–/–^ (*Ptgs1^–/–^*, indicated here as COX-1^–/–^) mice than in wild-type (COX-1^+/+^) (Figure 4, A and B). As expected, COX-1^–/–^ mice had decreased serum TXB_2_ levels (Figure 4C) and reduced platelet aggregation (Figure 4, D and E) compared with COX-1^+/+^ mice. Taken together, these data indicate that the inhibition of COX-1 is sufficient to impair metastasis development. They further suggest that inhibition of COX-1 mimics the antimetastatic effect of aspirin. These results led us to ask (a) what phase of metastasis is affected by inhibition of COX-1; (b) which product of COX-1 enables metastasis; and (c) which COX-1–expressing cells are responsible for these effects.

### Activity of COX-1 is required during the intravascular phase of metastasis.

**Figure 5:**
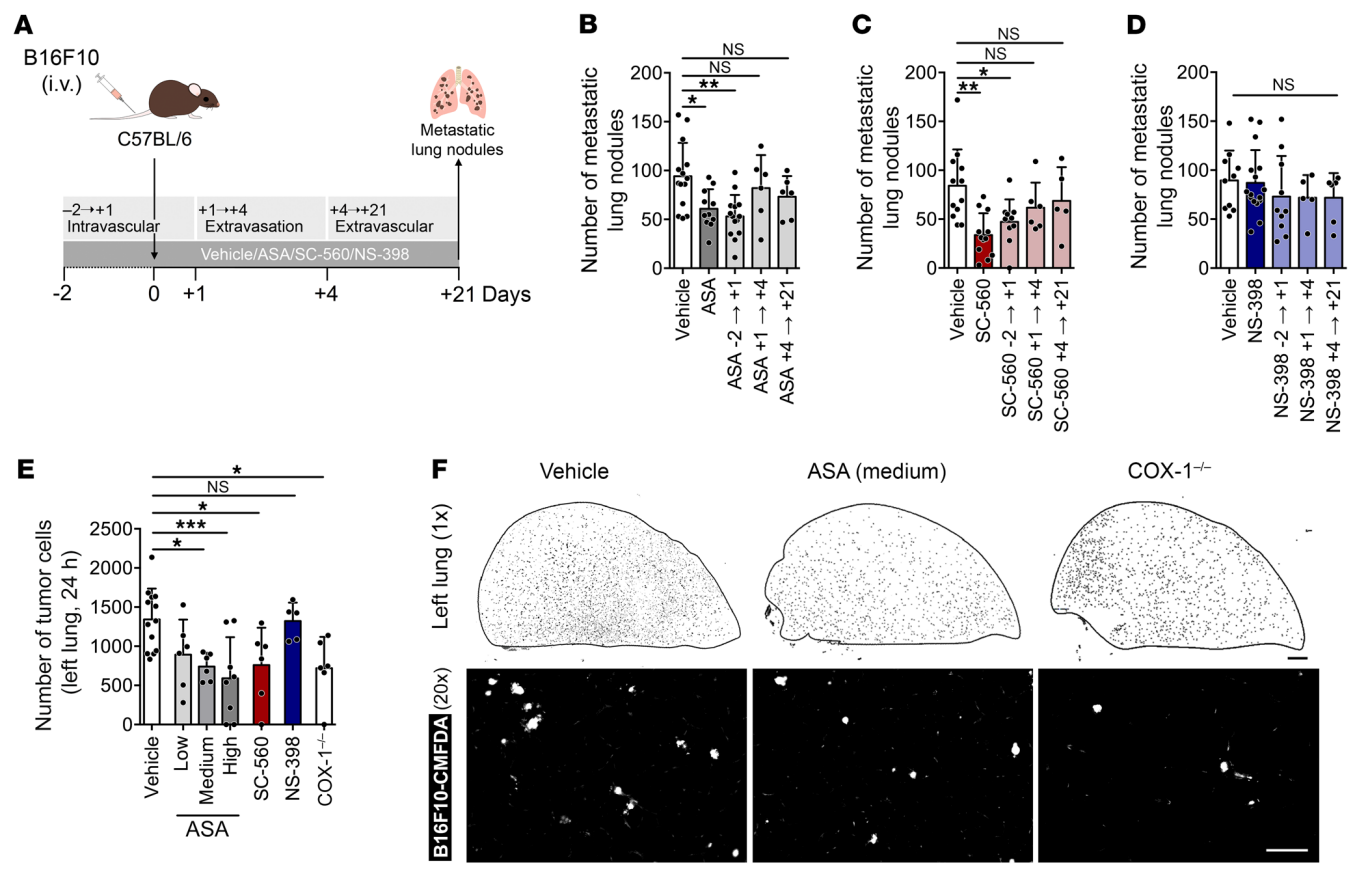
COX-1 inhibition affects the intravascular phase of metastasis. (**A**) Experimental design. (**B**–**D**) Number of B16F10 metastatic lung nodules in C57BL/6 mice treated with vehicle or aspirin (**B**), SC-560 (**C**), or NS-398 (**D**), according to **A**. (**B**) *n* = 14, 11, 14, 6, and 6; (**C**) *n* = 12, 12, 10, 6, and 5; (**D**) *n* = 10, 16, 10, 5, and 6. (**E** and **F**) Number of B16F10 cells (**E**) and representative tile scans (**F**; top panels, inverted look-up-table [LUT]; bottom panels, ×20 magnification) of the left lung of COX-1^–/–^ (*n* = 5) or of C57BL/6 mice treated with vehicle, aspirin (low, medium, and high doses), SC-560, or NS-398 (*n* = 12, 6, 6, 8, 6, and 5). Whole left lungs were imaged 24 hours after injection of B16F10-CMFDA cells (white). Scale bars: 1 mm (black bar), 100 μm (white bar). Data are represented as mean + SD. One-way ANOVA with Tukey’s multiple-comparisons test. *0.01 < *P* ≤ 0.05; **0.001 < *P* ≤ 0.01; ***P ≤ 0.001.

Disseminating tumor cells remain in the bloodstream for 1–4 days prior to extravasation, a time that varies depending on the model used (43). After tail vein injection, B16F10 cells were mainly intravascular after 24 hours and underwent extravasation between days 1 and 3 with the majority extravasated by day 4 (Supplemental Figure 5, A and B). The number of B16F10 cells rapidly decreased after injection, with only approximately 25% of the total adhered cells surviving in the lung vasculature 1 day after injection (Supplemental Figure 5C). Multicellular colonies were first noted on day 3, and micrometastases were seen on day 4 (Supplemental Figure 5, C and D). With day 0 being the day of injection, treatment was given from day –2 to day +1 (–2→+1) for the intravascular phase, from day +1 to day +4 (+1→+4) for the extravasation phase, or from day +4 to day +21 (+4→+21) for the extravascular phase (Figure 5A). Aspirin and SC-560 given during the intravascular phase of metastasis resulted in reduced numbers of metastatic lung nodules similar to treatment throughout. In contrast, administration during the later extravasation and extravascular phases did not affect metastasis (Figure 5, B and C). NS-398 did not change metastasis regardless of the schedule (Figure 5D). Aspirin and SC-560, but not NS-398, reduced the number of tumor cells in the lung 24 hours after injection (Figure 5, E and F). A similar reduction was obtained in COX-1^–/–^ mice (Figure 5, E and F). These findings suggest that COX-1 in host cells, rather than in tumor cells, is required for the pulmonary retention of tumor cells and the onset of metastasis.

### TXA_2_ signaling driven by COX-1 is essential for metastasis.

**Figure 6:**
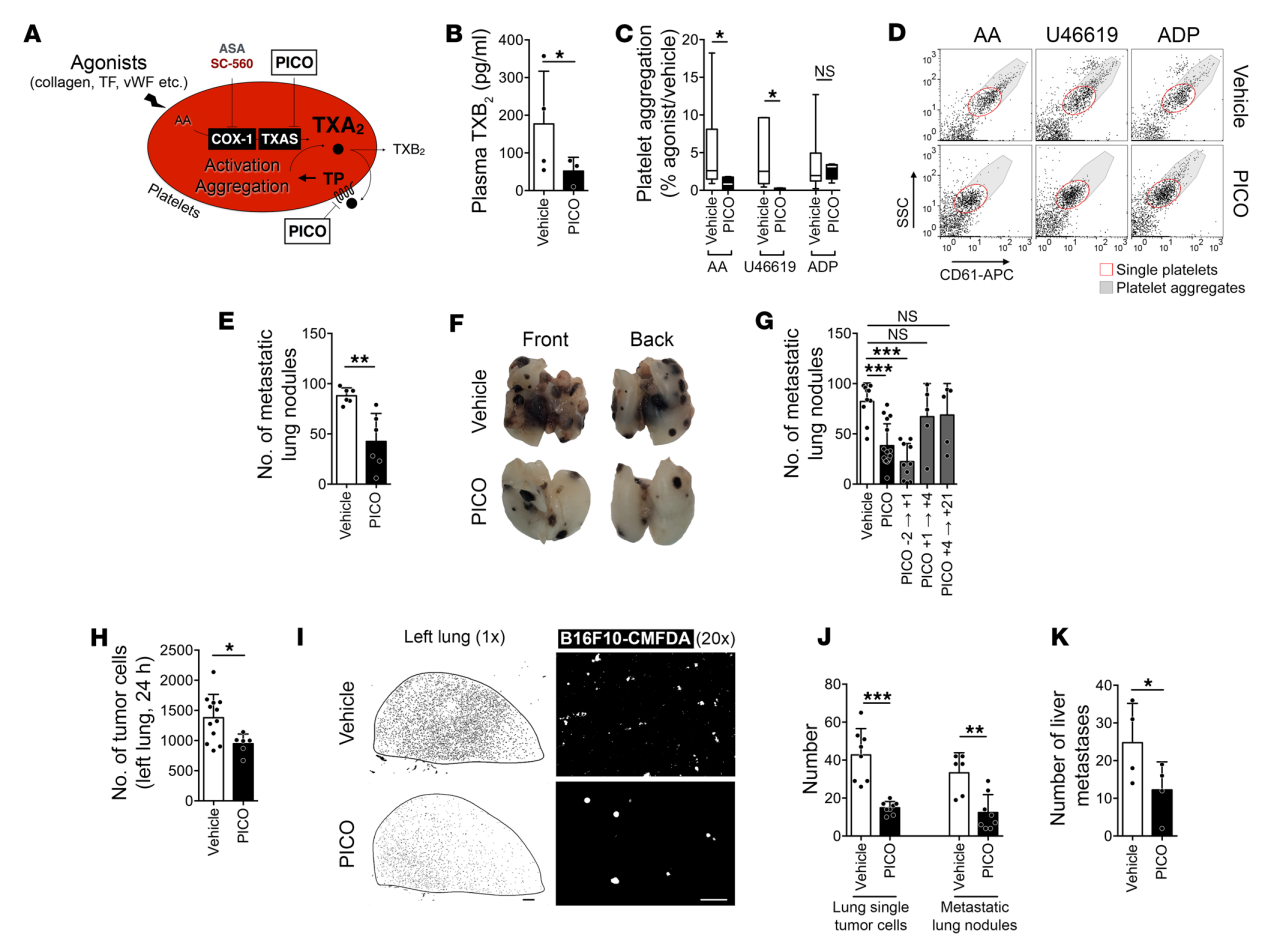
Inhibition of TXA_2_ signaling alters the intravascular phase of metastasis. (**A**) Diagram of the targets of picotamide (PICO) in platelets. (**B**) TXB_2_ in plasma from C57BL/6 mice treated with vehicle or picotamide for 2 days (*n* = 4). (**C** and **D**) Agonist-induced aggregation of platelets from C57BL/6 mice treated with vehicle or picotamide (*n* = 6 and 4). (**E** and **F**) B16F10 metastatic lung nodules in C57BL/6 mice treated with vehicle or picotamide (*n* = 6) as in Figure 2A. (**G**) B16F10 metastatic lung nodules in C57BL/6 mice treated with vehicle or picotamide, as in Figure 3A (*n* = 10, 14, 10, 5, and 5). (**H** and **I**) Number of B16F10-CMFDA cells (white) (**H**) and representative tile scans (**I**) of the left lung of C57BL/6 mice treated with vehicle or picotamide (*n* = 12 and 6) 24 hours after the injection of tumor cells. Scale bars: 1 mm (black bar), 100 μm (white bar). (**J** and **K**) Single disseminated cells in lungs (**J**) and metastatic nodules in lungs (**J**) or liver (**K**) of BALB/c mice bearing a 4T1-GFP tumor, treated with vehicle or picotamide (*n* = 8), as in Figure 2E. Control groups are the same as in Figure 2, F and H. Data are represented as mean + SD (**B**, **E**, **G**, **H**, **J**, and **K**), median ± range (**C**). Unpaired *t* test, 2-tailed (**B**, **E**, and **H**); 1-way ANOVA with Tukey’s multiple-comparisons test (**C**, **G**, **J**, and **K**). *0.01 < *P* ≤ 0.05; **0.001 < *P* ≤ 0.01; ***P ≤ 0.001.

Circulating TXA_2_ is the most abundant product of COX-1 in the circulatory system, mainly produced by activated platelets (4). To ask whether TXA_2_ was a critical intermediary in the COX-1–dependent development of metastasis, we administered picotamide (PICO), a dual inhibitor of TXAS and antagonist of TXA_2_ receptor (TP), to mice (Figure 6A) (44). This treatment reduced plasma levels of TXB_2_ (Figure 6B) and platelet aggregation (Figure 6, C and D), demonstrating inhibition of TXAS activity and TXA_2_/TP signaling. Treatment of mice with picotamide significantly reduced the number of B16F10 (Figure 6, E and F) and 4T1 (Supplemental Figure 3B) metastatic lung nodules. Picotamide treatment during the intravascular phase of metastasis (PICO –2→+1) reduced the number of metastatic lung nodules (Figure 6G) and decreased the early retention of tumor cells in the lungs (Figure 6, H and I). Furthermore, picotamide decreased the number of spontaneous metastases to liver and lungs, the number of disseminated tumor cells in the lungs (Figure 6, J and K), and the number and invasive capacity of CTCs (Supplemental Figure 4, B–E). Similarly, the TP antagonist vapiprost reduced platelet aggregation but not plasma TXA_2_ (Supplemental Figure 6, A–C), compatible with TP antagonism. Vapiprost impaired the early persistence of tumor cells in the lungs (Supplemental Figure 6, D and E), further supporting the notion that TXA_2_ signaling is required for early phases of metastatic seeding.

**Figure 7:**
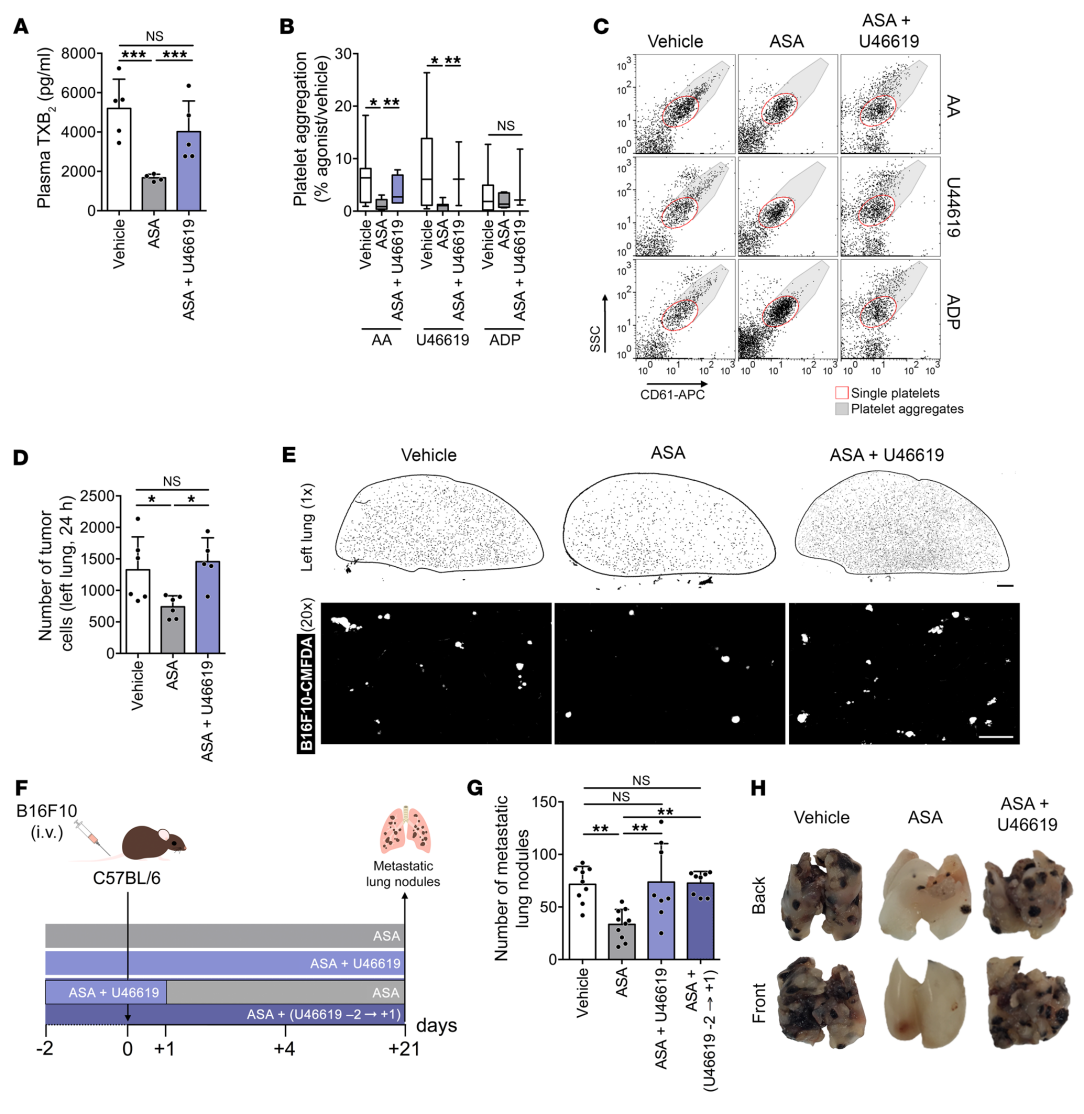
TXA_2_ analog U46619 abrogates the inhibition of metastasis by aspirin. (**A**) Plasma TXB_2_ in mice treated with vehicle, aspirin (medium dose), or aspirin supplemented with U46619 (ASA + U46619; *n* = 4). (**B** and **C**) Agonist-induced aggregation of platelets from mice treated with vehicle, aspirin, or aspirin + U46619 (*n* = 5, 4, and 5). (**D** and **E**) Number of tumor cells (**D**) and representative tile scans (**E**) from left lungs of mice treated with vehicle, aspirin, or aspirin + U46619 (*n* = 6, 6, and 5) 1 day after injection (B16F10-CMFDA, white). Scale bars: 1 mm (black bar), 100 μm (white bar). (**F**) Experimental design of aspirin with or without U46619 treatment. Two days before B16F10 cell injection, mice were treated with vehicle, aspirin, or aspirin + U46619 for 3 weeks (ASA + U46619) or supplemented until 1 day after injection, followed by treatment with aspirin alone [ASA + (U46619 –2→+1)]. (**G** and **H**) B16F10 metastatic lung nodules in mice treated with vehicle, aspirin, aspirin + U46619, or aspirin + (U46619 –2→+1) (*n* = 9, 10, 9, and 8). Data are represented as mean + SD (**A**, **D**, and **G**), median ± range (**B**). One-way ANOVA with Tukey’s multiple-comparisons test. *0.01 < *P* ≤ 0.05; **0.001 < *P* ≤ 0.01; ***P ≤ 0.001.

We then supplemented TXA_2_ using U46619, restoring basal levels of plasma TXA_2_/TXB_2_ (Figure 7A) and platelet aggregation (Figure 7, B and C) in aspirin-treated mice. This restored the numbers of persistent tumor cells in the lung to control values in aspirin-treated mice 1 day after injection (Figure 7, D and E) and of experimental metastases, even when U46619 was discontinued 1 day after tumor cell injection (Figure 7, F–H). These results suggest a central role for COX-1–derived TXA_2_ in the inhibition of metastasis by aspirin.

### TXA_2_ synthesis by COX-1 in platelets is required for metastasis.

**Figure 8:**
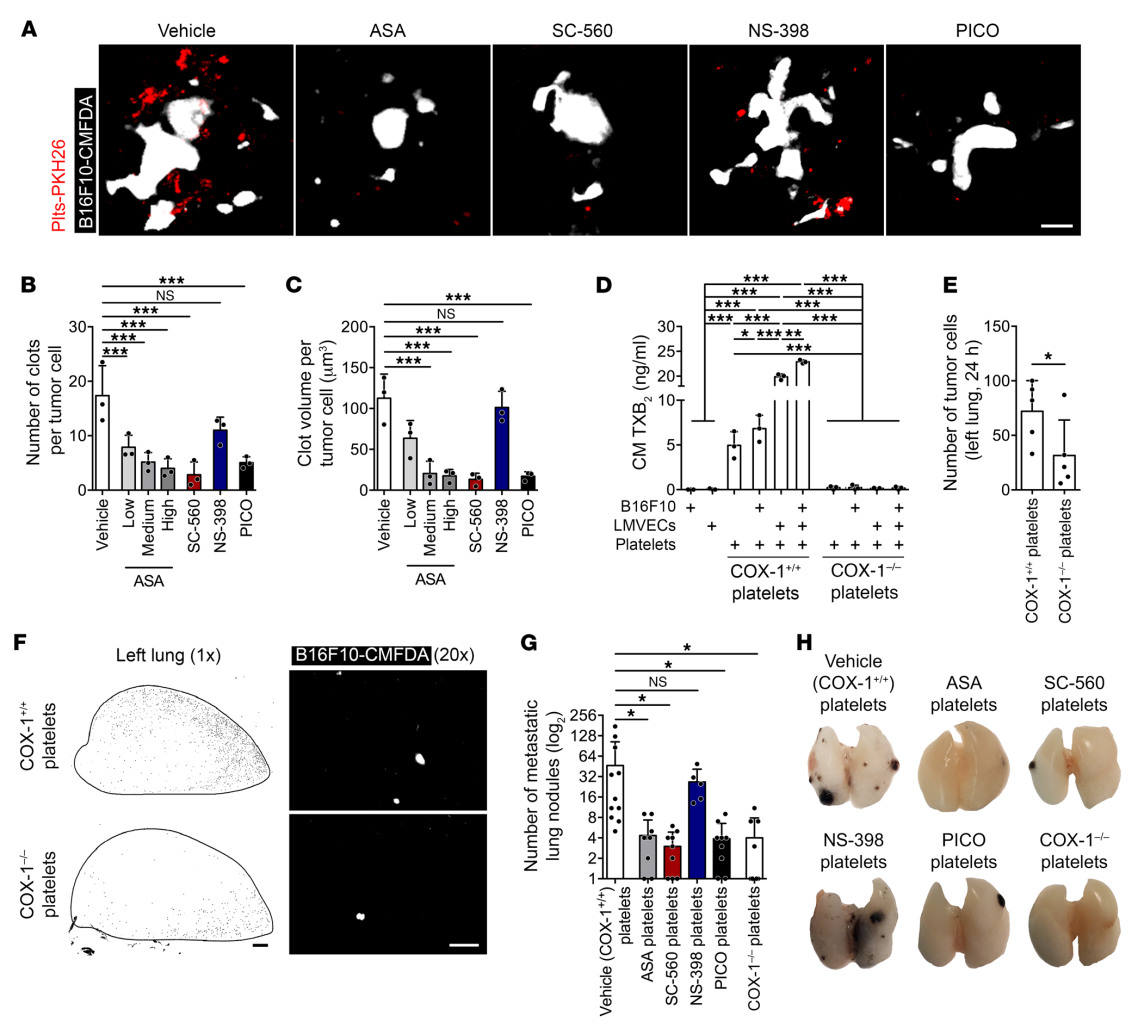
Platelet-derived TXA_2_ supports metastasis. (**A**–**C**) Maximum intensity projection (MIP, median filter) of 3D confocal stacks of tumor cells (B16F10-CMAC, white) and platelets (Plts-PKH26, red) and number (**B**) and volume (**C**) of platelet aggregates per tumor cells in whole lungs of *Cx3cr1^gfp/+^* mice, treated with vehicle, aspirin (low, medium, and high doses), SC-560, NS-398, or picotamide (*n* = 3). Lungs were harvested 8 hours after injection. Scale bar: 30 μm. (**D**) Concentration of TXB_2_ in conditioned medium (CM) from cultures of B16F10 cells, LMVECs, and/or COX-1^+/+^ or COX-1^–/–^ platelets (*n* = 3). (**E** and **F**) Number of tumor cells (**E**) and representative tile scans (**F**) of the left lung from platelet-depleted recipient mice (*n* = 5) at 1 day after i.v. injection of platelets (from COX-1^–/–^ or COX-1^+/+^ donor mice) and tumor cells (B16F10-CMFDA cells, white). Scale bars: 1 mm (black bar), 100 μm (white bar). (**G** and **H**) B16F10 metastatic lung nodules from platelet-depleted mice reinfused with COX-1 or COX-1^–/–^ platelets (*n* = 8) or COX-1^+/+^ platelets isolated from donor mice pretreated with vehicle, aspirin, SC-560, NS-398, or picotamide (*n* = 10, 9, 9, 5, and 9). Data are represented as mean + SD. One-way ANOVA with Tukey’s multiple-comparisons test (**B**–**D** and **G**); unpaired *t* test, 2-tailed (**E**). *0.01 < *P* ≤ 0.05; **0.001 < *P* ≤ 0.01; ***P ≤ 0.001.

TXA_2_ is synthesized by activated platelets and is a potent agonist of platelet aggregation and a secondary mediator of thrombus expansion (4). Platelets aggregate on the surface of B16F10 cells through a TF-dependent mechanism (27). We asked whether inhibition of COX-1 leading to reduced TXA_2_ levels results in decreased aggregation of platelets on tumor cells and reduced metastasis (8, 26, 45). Fluorescently labeled B16F10 cells (B16F10-CMAC) and platelets (Plts-PKH26) were injected into the opposite tail veins of mice. Platelet aggregation was observed only in the vicinity of the tumor cells, not distantly (Supplemental Figure 7A), suggesting that aggregation was triggered by the tumor cells. Additionally, platelets neither aggregated nor associated with the lung vasculature of naive mice (Supplemental Figure 7B), excluding the possibility that platelet aggregation resulted from euthanasia and its accompanying decreased blood flow. Treatment with aspirin, SC-560, and picotamide, but not NS-398, decreased the number and the size of clots per tumor cell (Figure 8, A–C). Similar results were obtained after coincubation of the 2 cell populations in vitro (Supplemental Figure 8, A–C). Pretreatment of platelets with aspirin and SC-560 diminished platelet aggregation on tumor cells, while pretreatment of tumor cells had no effect (Supplemental Figure 8, D–F). Additionally, COX-1^+/+^ platelets, but not COX-1^–/–^ platelets, B16F10 cells, nor primary lung microvascular endothelial cells (LMVECs) cells, generated TXB_2_ either alone or in coculture (Figure 8D). Thus, COX-1 in platelets associated with B16F10 cells is a major source of TXA_2_, and its inhibition affects platelet aggregation and thrombus expansion on tumor cells.

We then asked whether COX-1 inhibition in reintroduced platelets would reduce metastasis. After platelet depletion by R300 antibody, platelets isolated from vehicle-, ASA-, SC-560–, NS-398–, or PICO-treated mice or COX-1^–/–^ mice were infused after the antibody had dissipated but before platelets regained control levels (Supplemental Figure 9, A and B). Platelet depletion inhibited metastasis formation by B16F10 cells (Supplemental Figure 9C) (26, 45). The reinfusion of COX-1^+/+^ platelets in both COX-1^+/+^ (Figure 8, E and F) and COX-1^–/–^ (Supplemental Figure 9D) mice resulted in significantly greater pulmonary retention of tumor cells at 24 hours and enhanced numbers of metastatic lung colonies (Figure 8, G and H) compared with using COX-1^–/–^ platelets. Similarly, infusion of platelets from ASA-, SC-560–, and PICO-treated mice (Figure 8, G and H) or platelet-poor plasma (Supplemental Figure 9C) did not restore lung metastasis formation. Platelets from NS-398–treated mice restored metastatic colony formation (Figure 8, G and H). Together these results establish platelets as the COX-1/TXA_2_–dependent compartment in the establishment of metastasis.

### Inhibition of COX-1 reduces the adhesion of tumor cells to endothelium.

Tumor cell adhesion to endothelial cells during hematogenous metastasis involves multiple mechanisms (46) and seems to be facilitated by interactions with platelets (47). We investigated tumor cell adhesion to monolayers of LMVECs in the presence of platelets under flow with a low shear stress of 0.05 dyn/cm^2^. Firm tumor cell adhesion to LMVECs was measured after the flow was increased to a higher shear stress (1 dyn/cm^2^) (Supplemental Figure 10, A and B). Platelet aggregates adhered to tumor cells and formed bridges between tumor cells and LMVECs (Supplemental Figure 10C). Aspirin and SC-560 reduced the adhesion of tumor cells to LMVECs and the association of platelets with tumor cells (Supplemental Figure 10, D–F, and Supplemental Video 1). While the higher shear stress did not alter adhesion of the tumor cells, interestingly, it produced a significant dissociation of platelets from tumor cells under aspirin and SC-560 treatment (Supplemental Figure 10, G–I). These data suggest that COX-1 inhibition can reduce the adhesion of tumor cells to the endothelium.

### The COX-1/TXA_2_ axis in platelets contributes to an intravascular metastatic niche.

**Figure 9:**
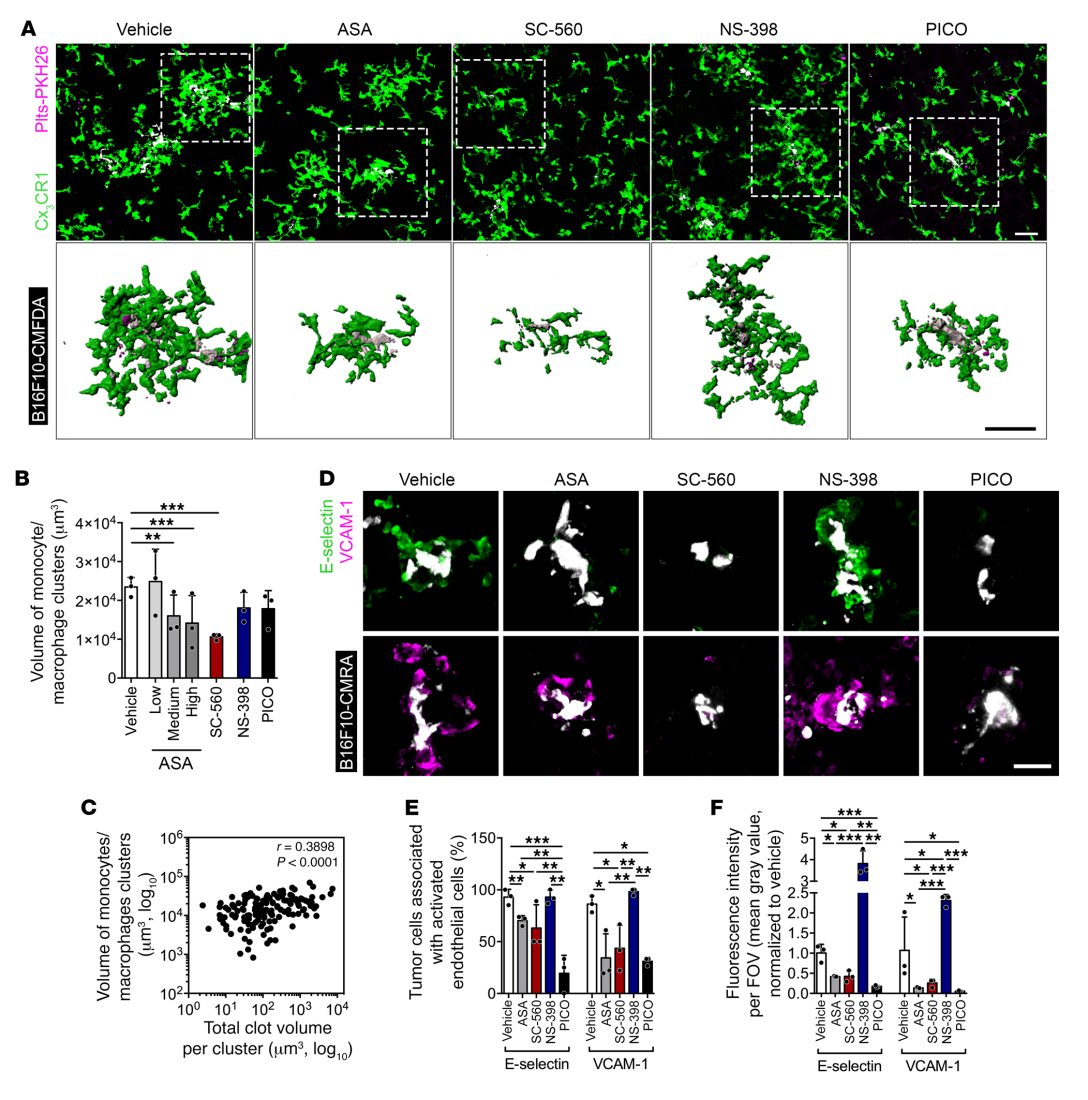
COX-1/TXA_2_ inhibition impairs the establishment of a permissive intravascular niche. (**A**) MIP (median filter) of 3D confocal stacks (×20, top row) and surface reconstruction (bottom row) of tumor cells (B16F10-CMAC, white), platelets (Plts-PKH26, magenta), and Cx_3_CR1^+^ monocytes/macrophages (GFP, green) in whole lungs of *Cx3cr1^gfp/+^* mice at 8 hours after injection of tumor cells and platelets. Scale bars: 50 μm. (**B**) Volume of monocyte/macrophage clusters (*n* = 3). (**C**) Correlation plot of the volume of monocyte/macrophage clusters versus the volume of clots within the cluster (*n* = 143). (**D**) MIP (median filter) of 3D confocal stacks of lung sections from *Cx3cr1^gfp/+^* mice treated with vehicle or drugs and injected with tumor cells (B16F10-CMRA, white). Activated endothelial cells were immunofluorescently labeled with an anti–E-selectin (green) or anti–VCAM-1 (magenta) antibody. Scale bar: 50 μm. (**E** and **F**) Number of tumor cells within an 80-μm radius from E-selectin– or VCAM-1–expressing vessels (**E**) (32) and fluorescence intensity of E-selectin or VCAM-1 (**F**) (*n* = 3). Data are represented as mean + SD. One-way ANOVA with Tukey’s multiple-comparisons test. *0.01 < *P* ≤ 0.05; **0.001 < *P* ≤ 0.01; ***P ≤ 0.001.

Microemboli are formed with tumor cells, platelets, and myeloid cells at sites of activated endothelium. The myeloid cells promote the survival of disseminating cells and their development into metastasis (27, 29, 32, 48). Using *Cx3cr1^gfp/+^* mice to visualize monocytes and macrophages (27, 49), we found that aspirin (medium and high doses) and SC-560, but not NS-398 and picotamide, reduced clustering of monocytes/macrophages around the intravascular tumor cells (Figure 9, A and B). The magnitude of monocyte recruitment correlated with the extent of the platelet clots (Figure 9C). Treatment with aspirin, SC-560, and picotamide also reduced the extent of endothelial activation as indicated by E-selectin and VCAM-1 expression in vessels adjacent to platelet–tumor cell aggregates (Figure 9, D–F). Neither monocyte/macrophage recruitment nor endothelial activation was observed in naive mice (Supplemental Figure 7). Additionally, inhibition of COX-1/TXA_2_ was associated with a larger diameter of lung vessels (Supplemental Figure 11, A–C), suggesting a decrease of vasoconstriction that might further prevent the accumulation of aggregates.

**Figure 10:**
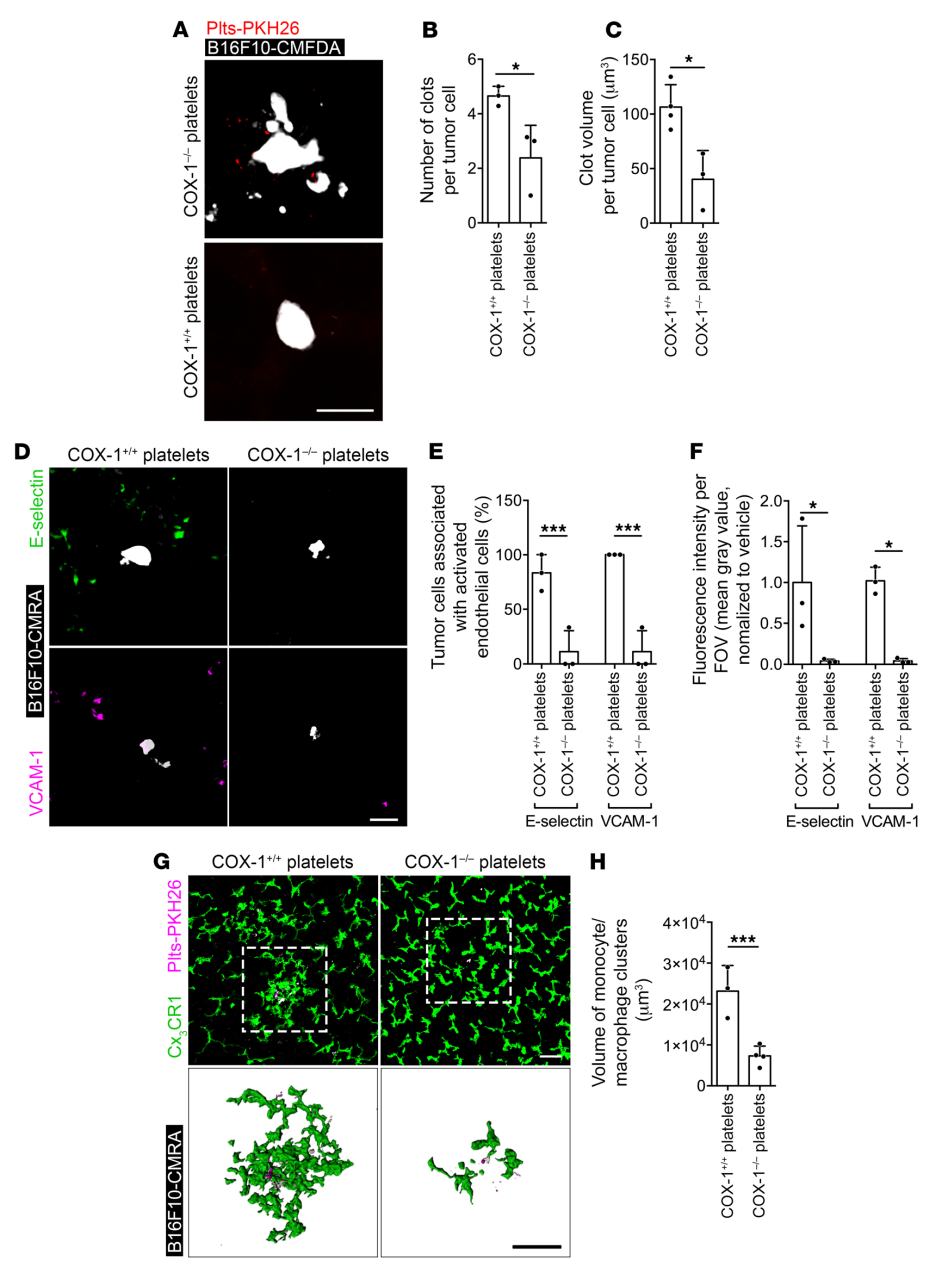
TXA_2_ from platelets mediates the generation of the prometastatic intravascular niche. (**A**–**C**) MIP (median filter) of 3D confocal stacks of tumor cells (B16F10-CMAC, white) and platelets (Plts-PKH26, red) in lungs of platelet-depleted *Cx3cr1^gfp/+^* mice (**A**) and quantification of the number (**B**) and volume (**C**) of clots per tumor cell (*n* = 3), at 8 hours after injection of tumor cells and COX-1^+/+^ or COX-1^–/–^ platelets. Scale bar: 10 μm. (**D**–**F**) MIP (median filter) of 3D confocal stacks of lung sections labeled for E-selectin (green) and VCAM-1 (magenta) (**D**), number of tumor cells associated with E-selectin– or VCAM-1–expressing vessels (**E**), and fluorescence intensity of E-selectin or VCAM-1 (**F**) (*n* = 3) in lung sections from platelet-depleted *Cx3cr1^gfp/+^* mice injected with tumor cells and COX-1^+/+^ or COX-1^–/–^ platelets. Scale bar: 50 μm. (**G** and **H**) MIP of 3D confocal stacks (×20, top row) and surface reconstruction (bottom row) of tumor cells (B16F10-CMAC, white), platelets (Plts-PKH26, magenta), and Cx_3_CR1^+^ monocytes/macrophages (GFP, green) (**G**) and quantification of the volume of monocyte/macrophage clusters (**H**) (*n* = 3) in whole lungs of platelet-depleted *Cx3cr1^gfp/+^* mice injected with tumor cells and COX-1^+/+^ or COX-1^–/–^ platelets. Scale bars: 50 μm. Data are represented as mean + SD. Unpaired *t* test, 2-tailed. *0.01 < *P* ≤ 0.05; ***P ≤ 0.001.

Analogous effects resulted from coinfusion of COX-1^–/–^ platelets and B16F10 cells in COX-1^+/+^ mice, with a decrease in platelet aggregation on tumor cells (Figure 10, A–C), association of tumor cells with activated endothelium (Figure 10, D–F), diameter of blood vessels (Supplemental Figure 11, D–F), and recruitment of monocytes/macrophages to tumor cells (Figure 10, G and H) in comparison with mice infused with COX-1^+/+^ platelets.

### The COX-1/TXA_2_ pathway contributes to a pulmonary premetastatic niche.

**Figure 11:**
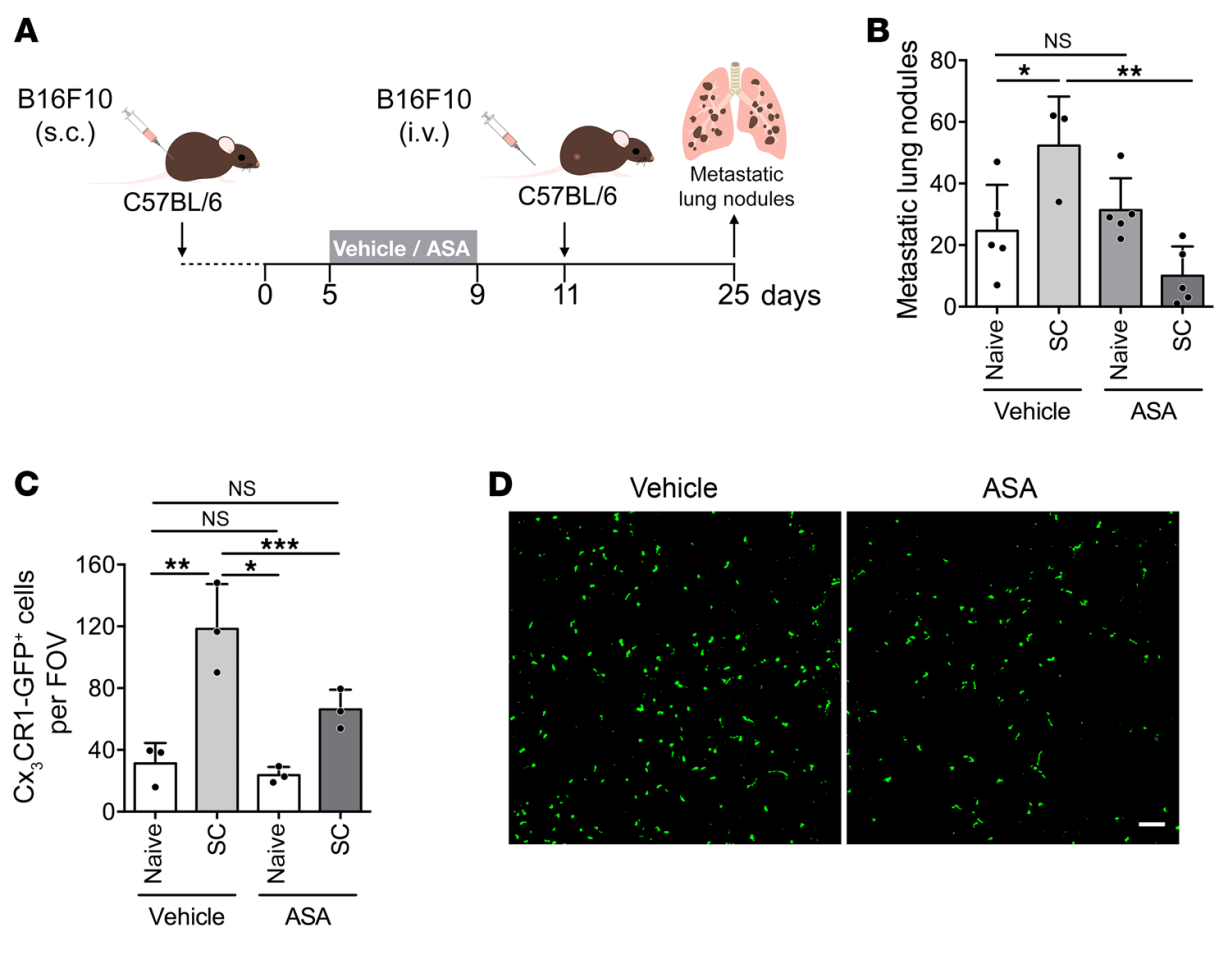
The COX-1/TXA_2_ pathway is required for the establishment of the premetastatic niche. (**A**) Experimental design. (**B**) Number of B16F10 metastatic lung nodules in C57BL/6 mice bearing no tumor (naive) or subcutaneous B16F10 tumor (SC), treated with vehicle or aspirin (medium dose) as in **A** (*n* = 5, 3, 5, and 5). (**C** and **D**) Numbers of GFP^+^ monocytes/macrophages per field of view (FOV) in naive or SC *Cx3cr1^gfp/+^* mice treated with vehicle or aspirin (**C**), and representative confocal images of lung sections from mice (**D**). Lungs were harvested 11 days after s.c. inoculation of tumor cells, before i.v. injection of B16F10 cells (*n* = 3). Scale bar: 200 μm. Data are represented as mean + SD. One-way ANOVA with Tukey’s multiple-comparisons test. *0.01 < *P* ≤ 0.05; **0.001 < *P* ≤ 0.01; ***P ≤ 0.001.

The ability of disseminated tumor cells to colonize distant sites is enhanced by the systemic effects of a primary tumor, generating a premetastatic niche (50). To test the effect of inhibition of the COX-1/TXA_2_ pathway on the establishment of a lung premetastatic niche, mice bearing B16F10 subcutaneous tumors were treated with aspirin and injected i.v. with tumor cells to induce lung metastasis before the occurrence of spontaneous metastasis (Figure 11A). Aspirin treatment was started after the initiation of tumor growth and interrupted 2 days before tumor cell injection to avoid a direct effect of platelet inhibition on metastatic seeding. The increased numbers of metastatic lung nodules, indicative of the establishment of a premetastatic niche, were completely abrogated by treatment with aspirin (Figure 11B). Aspirin did not affect the number of nodules in mice without subcutaneous tumors (Figure 11B), further supporting the prometastatic role of intact COX-1/TXA_2_ axis in platelets at the moment of tumor cell injection.

Lung preconditioning has been linked to the recruitment of myeloid cells with the support of the coagulation system (27, 51, 52). The numbers of Cx_3_CR1-GFP^+^ monocytes/macrophages in the lungs of mice bearing tumors were greater than those in lungs of naive mice. Aspirin abolished this increase in monocytes/macrophages in the premetastatic lungs (Figure 11, C and D) but did not affect the numbers in naive mice (Figure 11C). Taking into account the role of platelets in the recruitment of myeloid cells (27, 29, 33) and the effect of the TXA_2_ inhibitor picotamide on the establishment of spontaneous metastasis (Figure 6, J and K), together these data suggest that the establishment of a lung premetastatic niche depends on the COX-1/TXA_2_ pathway in platelets.

### TXA_2_ signaling, not other platelet activation pathways, is required for the establishment of the intravascular metastatic niche.

**Figure 12:**
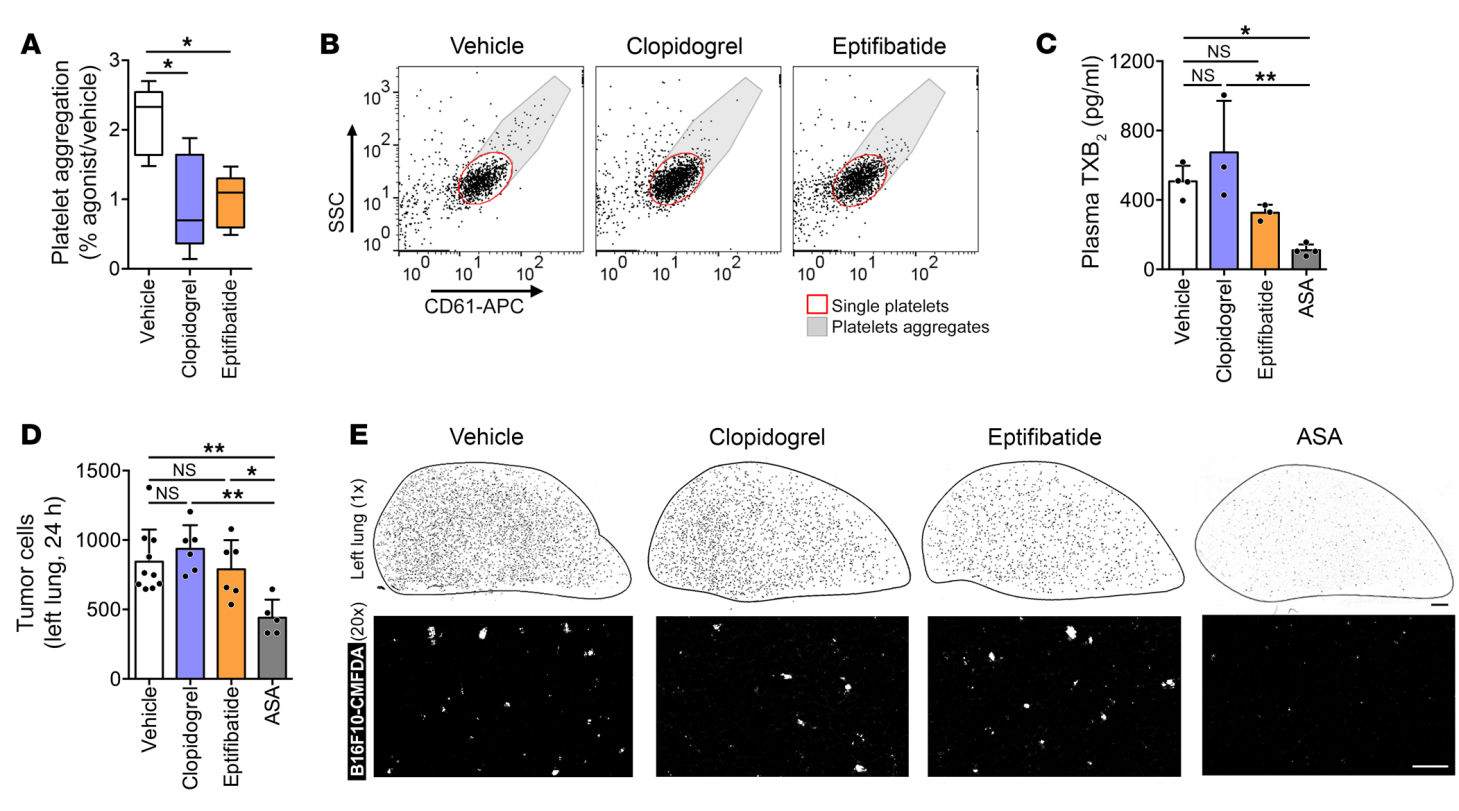
Platelet activation pathways other than COX-1/TXA_2_ are not required for early metastatic seeding. (**A** and **B**) ADP-induced aggregation of CD61-stained platelets from C57BL/6 mice treated with vehicle, clopidogrel, or eptifibatide for 2 days (*n* = 4). (**C**) TXB_2_ in plasma from mice treated with vehicle or drugs for 2 days (*n* = 4, 3, 3, and 4). (**D** and **E**) Number of B16F10-CMFDA cells (white) (**D**) and representative tile scans (**E**) of the left lung of C57BL/6 mice treated with vehicle or drugs (*n* = 10, 6, 6, and 5), imaged at 24 hours after the injection of tumor cells. Scale bars: 1 mm (black bar), 100 μm (white bar). Data are represented as median ± range (**A**), mean + SD (**C** and **D**). One-way ANOVA with Tukey’s multiple-comparisons test. *0.01 < *P* ≤ 0.05; **0.001 < *P* ≤ 0.01.

To understand whether platelet aggregation generally is critical for creating a metastatic niche or whether TXA_2_ signaling is more specifically required, we tested clopidogrel, an antagonist of the P2Y12 ADP purinergic receptor, and eptifibatide, an inhibitor of α_IIb_β_3_ integrin (also known as GPIIb/IIIa), both used clinically to reduce platelet aggregation (53–56). Clopidogrel and eptifibatide significantly reduced ADP-induced platelet aggregation (Figure 12, A and B) without affecting plasmatic TXB_2_ levels in vivo (Figure 12C), compatible with a functional COX-1/TXA_2_ pathway in platelets. Unlike aspirin, clopidogrel and eptifibatide did not affect the early persistence of B16F10 melanoma cells in the lungs (Figure 12, D and E), suggesting that TXA_2_ signaling in the context of platelet aggregation is essential for the establishment of the early metastatic niche.

**Figure 13:**
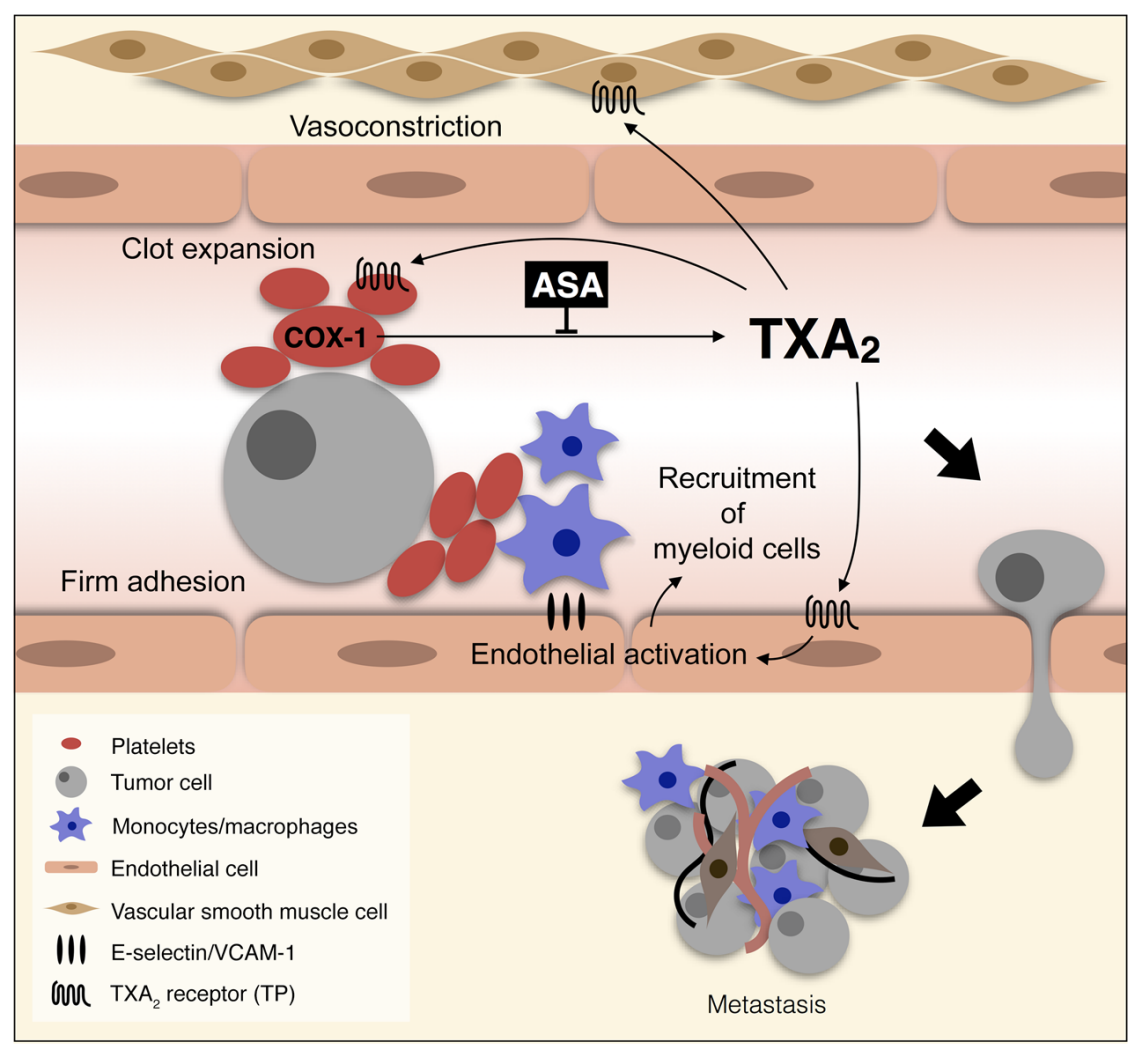
Platelet-derived TXA_2_ promotes metastasis by generating a permissive intravascular metastatic niche. Model of metastasis promotion by TXA_2_ derived from COX-1 activity in platelets. The aggregation of platelets on tumor cells stimulates the aspirin-sensitive de novo synthesis of TXA_2_, which enhances the expansion of clots on tumor cells and leads to further TXA_2_ synthesis. Concomitantly, TXA_2_-TP interaction induces the contraction of vascular smooth muscle cells, the activation of endothelial cells, and the recruitment of monocytes/macrophages to tumor cells, providing a permissive niche for metastasis seeding.

All together our data describe a signaling network centered on platelet-derived TXA_2_ that can be inhibited by aspirin treatment, leading to a reduced seeding efficiency and metastasis (Figure 13).

## Discussion

In this paper we have provided evidence that aspirin reduces metastasis through the inhibition of platelet COX-1 and its product TXA_2_. Inhibition of COX-1 activity or TXA_2_ signaling alone by pharmacological or genetic means was sufficient to reduce metastasis in a range of models. This novel finding directly implicates the activity of COX-1/TXA_2_ in platelets before and during the intravascular transit of tumor cells, while it is not necessary for the persistent growth of the metastatic lung nodules. The inhibition of COX-1/TXA_2_ in platelets impairs multiple consecutive steps of the hematogenous transit of tumor cells, leading to the reduction of tumor cells in the lung vasculature. Thus, COX-1 activity and TXA_2_ production in platelets contribute to the generation of a permissive early metastatic niche (Figure 13).

Aspirin has distinctive pharmacological properties at different doses, mainly derived from the differential inhibition of COXs in different body compartments. The antimetastatic effect of aspirin was seen at doses that inhibited COX-1/TXA_2_, whereas the inhibition of COX-2/PGE_2_ alone was not sufficient, suggesting a prominence of COX-1 rather than COX-2 in the metastatic process. We exclude COX-independent targets (57) since analogous results were obtained with inhibitors of other steps in the COX pathway and in COX-1^–/–^ mice. The antimetastatic effect of aspirin was seen in low- and medium-dose trials (75–300 mg/d), and increased doses did not show additional benefit (23), consistent with platelet COX-1 as the main target for the antimetastatic effect of aspirin. To the best of our knowledge, COX-1 has been previously implicated only marginally in the development of metastasis (58). COX-1 can be expressed by a variety of cell types (21). Reinfusion of platelets in platelet-depleted mice only restored metastasis if the platelets contained active COX-1, showing that it is the platelet supply of COX-1 that is essential to metastasis.

Further, although COX-1 can generate a variety of active prostaglandins, the reduction of TXA_2_ is responsible for the antimetastatic effect of aspirin. Infusion of a synthetic analog of TXA_2_ restored the metastatic phenotype during aspirin treatment. Platelets aggregate on the surface of tumor cells and function as circulating reservoirs of TXA_2_. Autocrine TXA_2_ signaling in platelets further enhances their aggregation on tumor cells, which supports metastasis (8, 26, 45). Additionally, paracrine TXA_2_ signaling generates a favorable environment for tumor cell seeding through vascular constriction and induction of E-selectin and VCAM-1 through the TP receptor on endothelial cells (59, 60). Cytokines released from intracellular granules of activated platelets also induce endothelial cell activation (61). Endothelial activation correlates with tumor cell survival within the lung vasculature (32), and E-selectin and VCAM-1 might facilitate tumor cell adherence to the endothelium directly (62) or via bound platelets (63). We demonstrated enhanced adhesion of tumor cells to an LMVEC monolayer in the presence of platelets, analogous to the results in vivo. Concomitantly, endothelial activation facilitates the homing and retention of metastasis-promoting monocytes/macrophages in proximity to the tumor cells (32, 61). Monocyte chemoattractant protein-1 (CCL2/MCP-1) and CCL5 release by endothelial cells following TXA_2_ signaling might amplify recruitment (29, 64). Altogether, local release of TXA_2_ leads to the formation of hematogenous microemboli with prometastatic properties. The recruitment of monocytes/macrophages was also reduced by aspirin at the level of the premetastatic niche, leading to reduced lung seeding. These data support the notion that cancer-induced thrombosis via the COX-1/TXA_2_ pathway plays a central role in the conditioning of metastatic sites both before and after the arrival of CTCs (27).

The inhibition of COX-2 decreases metastasis in some models (10, 11) but not others (12). In our experiments, NS-398 did not reduce seeding of B16F10- and 4T1-derived lung metastasis, but it decreased the size of metastatic lung nodules from B16F10 cells, consistent with COX-2 enhancing proliferation and immune evasion in experimental models (65, 66). We noted that inhibition of COX-2 significantly inhibited metastasis by one colorectal cancer cell line, MC-38-GFP. Some colorectal cancers depend on COX-2 for progression (66), and we confirmed that MC-38 cells express much higher levels of COX-2 than B16F10 cells (S. Lucotti, unpublished observations). Thus, the sensitivity to COX-2 inhibition might be indicated by COX-2 expression in cancer cells (67).

In contrast, our data point to COX-1 inhibitors reducing metastasis through a microenvironment-centered mechanism. COX-1 inhibition was effective on cell lines regardless of their COX-1 expression (S. Lucotti, unpublished observations), and COX-1^–/–^ mice had reduced metastasis, suggesting that aspirin has an antimetastatic effect independent of tumor cell expression of COX-1. Using Oncomine gene expression data we found that COX-1 expression in the primary tumor did not correlate with risk of metastatic cancer nor with the antimetastatic effect of aspirin (Supplemental Figure 12). These data are consistent with the hypothesis that tumor cells with higher procoagulant activity are more sensitive to the antimetastatic effect of COX-1 inhibition (11, 24). We showed that aspirin only at doses inhibiting COX-1 and specific COX-1 inhibitors blocked metastasis in 4 different metastasis models as well as spontaneous metastasis by 4T1. A limitation of our study, however, is that we only detailed the mechanisms for B16F10 melanoma cells. We could hypothesize that a similar inhibition of platelet COX-1 would occur in a range of models.

It is well established that platelet function greatly supports metastatic spread (68). Pharmacological or genetic inhibition of P2Y12 receptor and α_IIb_β_3_ integrin has been previously found to be associated with reduced numbers of experimental metastasis (53, 55, 56). We failed to find any effect of the P2Y12 receptor antagonist clopidogrel and the α_IIb_β_3_ integrin inhibitor eptifibatide on the early metastatic seeding of B16F10 cells. These findings suggest that alternative pathways of platelet aggregation are not required for the establishment of the intravascular metastatic niche, but they do not exclude the possibility that these pathways support later stages of metastasis such as epithelial-mesenchymal transition and extravasation (55, 69).

Overall, this work identifies COX-1 and its product TXA_2_ as potential pharmacological targets to inhibit the intravascular phase of metastasis, consistent with the use of low- to medium-dose aspirin as adjuvant therapy for cancer patients. The recently started phase III ADD-ASPIRIN Trial (70) will examine the effects of aspirin in the prevention of tumor relapse and metastasis. However, aspirin significantly increases the risk of severe gastrointestinal symptoms and complications, especially over long-term use. Our data, together with previous clinical trials (71–73), suggest that selective TXA_2_ inhibitors such as picotamide might present an alternative to target platelet TXA_2_ while sparing other gastroprotective COX-1 products (i.e., PGI_2_), and thus might be a safer therapeutic option for the prevention of metastatic disease.

## Methods

### Animals.

C57BL/6 (C57BL/6J), BALB/c (BALB/cAnNCrl), and SCID (CB17/Icr-Prkdc^scid^/IcrIcoCrl) mice were purchased from Charles River Laboratories and *Cx3cr1^gfp/+^* mice (B6.129P-*Cx3cr1^tm1Litt^*/J) from The Jackson Laboratory (49). COX-1^–/–^ mice (74) were provided by TDW and JAM. Seven- to ten-week-old female mice were used for experiments involving drug treatment and/or tumor cell injection, while older naive mice with a C57BL/6 background were used for blood withdrawal and platelet isolation. Drugs were administered through drinking water, given ad libitum and changed every second day.

### Cell lines and staining.

B16F10 murine melanoma cells (a gift from John L. Francis, Center for Thrombosis Research, Florida Hospital, Orlando, Florida, USA; ref. 75) were cultured in RPMI 1640 medium (Sigma-Aldrich), 4T1/4T1-GFP murine breast cancer cells, MC-38-GFP murine colorectal cancer cells, and MDA-MB-231-CFP human breast cancer cells (ATCC) were cultured in DMEM (Sigma-Aldrich) in a 5% CO_2_ humidified atmosphere at 37°C. Media were supplemented with 10% heat-inactivated FBS (Gibco), 2 mM l-glutamine, 25 mM HEPES, 50 U/ml penicillin, and 5 μg/ml streptomycin (Thermo Fisher Scientific), with addition of 0.4 mg/ml G418 or 5 μg/ml puromycin for 4T1-GFP and MC-38-GFP cells, respectively. Primary LMVECs were cultured in 2% gelatin–coated flasks (Sigma-Aldrich) in enriched DMEM (76). Cells were passaged using Versene (B16F10) (Thermo Fisher Scientific) or 0.05% trypsin-EDTA solution (all other cell lines) (Sigma-Aldrich). LMVECs were used within 10 and tumor cells within 20 passages and routinely tested for mycoplasma contamination (MycoAlert Mycoplasma Detection Kit, Lonza Group Ltd.). Exponentially growing B16F10 cells (50%–60% confluence) were stained with 12.5 μM solution of CellTracker Blue CMAC, Orange CMRA, or Green CMFDA dye (Thermo Fisher Scientific), following the manufacturer’s instructions.

### Drug formulation for animal studies.

Aspirin (ASA), purchased as dl-lysine acetylsalicylate (Aspégic injectable, Sanofi Aventis), was dissolved in sterile deionized water and resuspended in drinking water at 30 mg/l (low) (37), 180 mg/l (medium) (38), or 625 mg/l (high) (8). SC-560 (Cayman Chemical) dissolved in DMSO (Sigma-Aldrich) was resuspended at 24 mg/l in drinking water supplemented with 0.2% (vol/vol) polyethylene glycol 200 (PEG200) and 0.01% (vol/vol) Tween-20 (both from Sigma-Aldrich) (77). NS-398 (Cayman Chemical) dissolved in DMSO was resuspended at 12 mg/l in drinking water supplemented with 0.9% wt/vol sodium chloride (Sigma-Aldrich) (77). Picotamide (PICO; Abcam) dissolved in 100% ethanol (Sigma-Aldrich) was resuspended in drinking water at 30 mg/l. U46619 (Cayman Chemical) was diluted in DMSO and delivered at 50 μg/kg through a 180-mg/l aspirin solution. Vapiprost (Vapiprost hydrochloride, Santa Cruz Biotechnology) dissolved in sterile water was resuspended in drinking water at 20 mg/l. All drinking water contained 2% wt/vol sucrose (Sigma-Aldrich). Clopidogrel [(±)-Clopidogrel (hydrochloride), Cayman Chemical] dissolved in 100% ethanol was delivered in saline at 10 mg/kg/d through i.p. injection (56). Eptifibatide (Sigma-Aldrich) was resuspended in sterile water and delivered in saline at 0.5 mg/kg/d through i.p. injection (78).

### Isolation and staining of platelets.

After sacrifice with an overdose of pentobarbital (665 mg/kg, i.p., or 332.5 mg/kg, i.v.), blood was collected from mice by cardiac puncture in syringes containing 3.2% (wt/vol) sodium citrate (Thermo Fisher Scientific) or ACD buffer (83 mM Na_3_C_6_H_5_O_7_, 111 mM dextrose, 71 mM citric acid) (Sigma-Aldrich and Fisher Thermo Scientific), at 1:10 vol/vol ratio to blood.

To test aggregation, citrated blood was diluted 1:2 with modified Tyrode’s-HEPES (MTH) buffer (134 mM NaCl, 0.3 mM NaH_2_PO_4_•2H_2_O, 3 mM KCl, 5 mM HEPES, 5 mM dextrose, 2 mM MgCl_2_) (Sigma-Aldrich and Fisher Thermo Scientific) supplemented with 0.02 U/ml apyrase (Sigma-Aldrich) and 0.25 μM PGE_1_ (Alprostadil, Sigma-Aldrich). After centrifugation at 180 *g* for 10 minutes at 22°C, the supernatant was collected (platelet-rich plasma [PRP]). The remaining pellet was centrifuged at 12,000 *g* for 2 minutes at room temperature, and the supernatant was collected (platelet-poor plasma [PPP]).

To prepare washed platelets, PRP was diluted with washing buffer (10% MTH vol/vol in dH_2_O, 0.10% wt/vol NaHCO_3_, 0.20% wt/vol BSA, and 1 mM EGTA) and centrifuged at 1300 *g* at 22°C for 10 minutes. The platelet pellet was washed twice with washing buffer containing 0.25 μM PGE_1_. Platelets were counted in a Coulter counter (Beckman; 50-μm aperture tube, 3–30 fl particles). Washed platelets (8 × 10^5^ cells/μl) were stained with PKH26 (Sigma-Aldrich) and readjusted to the required concentration in PPP or resuspension buffer (10% MTH vol/vol in dH_2_O, 0.10% wt/vol NaHCO_3_, and 0.20% wt/vol BSA).

### Platelet aggregometry.

Platelet aggregation was evaluated as previously described (79). Briefly, citrated PRP was incubated with agonists arachidonic acid (1 μM; Sigma-Aldrich), U46619 (0.3 μM; Tocris), and ADP (1 M; ChronoLog) or their vehicles in half-area-96-well microtiter plates for 5 minutes at 37°C under 1 mm orbital shaking (Infinite m200 plate reader, Tecan). Aggregated PRP was diluted 1:4 with ACD buffer and labeled with anti-CD61–APC antibody (104316, BioLegend) for 30 minutes at 4°C. Samples were then diluted with 0.01% neutral buffered formalin in PBS (Sigma-Aldrich) and supplemented with 10^4^ CountBright absolute counting beads (Thermo Fisher Scientific). The suspension was analyzed with a FACSCalibur flow cytometer (BD Biosciences). Beads (FL1/SSC-H) were gated and platelets (FL4-H/SSC-H) were acquired until the count of 100 beads was reached. The total number of single platelets was calculated using FlowJo software (version 7.6.5). Depletion of the single-platelet population is representative of platelet aggregation, and can be visualized through the appearance of a comet tail of platelet aggregates (79).

### Ex vivo platelet aggregation on tumor cells.

CMFDA-stained B16F10 cells were seeded at 10^4^ cells per well in collagen I–biocoated multichambers (BD Biosciences). The following day, 30 × 10^6^ PKH26-stained platelets were added together with vehicle or drugs. After 2 hours, cells were fixed with 2% paraformaldehyde (PFA) in PBS and mounted with Vectashield mounting medium containing DAPI (Vector Laboratories). Tumor cells and platelets were pretreated with vehicle or drugs for 2 hours at 37°C or 30 minutes at 30°C, respectively, washed twice with PBS or washing buffer, and coincubated.

### Experimental lung metastasis assay.

B16F10 (2.5 × 10^5^) and MC-38-GFP (3.0 × 10^5^) cells were injected i.v. into C57BL/6 mice, 4T1 (1.5 × 10^5^) into BALB/c mice, and MDA-MB-231-CFP (1 × 10^6^) into SCID mice. R300 antibody (0.5 mg/kg; Emfret) for platelet depletion or isotype control (C301, Emfret) was injected i.p. 24 hours before i.v. injection of tumor cells and PPP (<1 × 10^6^ platelets), PRP, or platelets (6 × 10^8^ platelets). After 2 weeks (4T1 cells), 3 weeks (B16F10 and MC-38-GFP cells), or 4 weeks (MDA-MB-231-CFP cells), mice were anesthetized (pentobarbital, 70 mg/kg, i.p.) and their lungs artificially ventilated through a tracheotomy. After sacrifice, lungs were perfused through the pulmonary artery with Krebs-Ringer buffer (KRB) (4.74 mM KCl, 1.17 mM MgSO_4_•7H_2_O, 1.27 mM CaCl_2_•2H_2_O, 1.18 mM KH_2_PO_4_, 118.4 mM NaCl, 24.87 mM NaHCO_3_, 10 mM dextrose, 5% wt/vol dextran) (Sigma-Aldrich or Thermo Fisher Scientific). After clearance of blood, lungs were immersed in 10% neutral buffered formalin (Sigma-Aldrich). Metastatic lung nodules were visually counted (B16F10, MC-38-GFP, and 4T1 cells) or assessed by MRI scan (MDA-MB-231-CFP cells). Lobes were embedded in paraffin, and 5-μm sections were stained by H&E.

### MRI scan of lungs.

MRI on formalin-fixed lungs embedded in 4% agarose was performed at 4.7T or 7.0T (VNMRS, Agilent) using a 25-mm-inner-diameter quadrature birdcage coil (Rapid). T2-weighted fast spin echo 3D scan was acquired (echo spacing 9.35 ms, echo train length 8, effective echo time [TE] 37.41 ms, repetition time [TR] 200 ms) with a field of view (FOV) of 32 × 32 × 32 mm^3^ to ensure complete coverage of the coil (and sample). Scan time was approximately 27 minutes per sample for an isotropic resolution of 125 μm. Ten samples were queued for unsupervised MRI measurement using an in-house-developed carriage system utilizing a stepper motor driven by an Arduino controller (http://www.arduino.cc). Tumor burden was quantified by manual segmentation via ImageJ (version 1.46r, NIH) and itk-SNAP software (version 3.6.0) (80).

### Ex vivo whole lung imaging assay.

CMAC-stained B16F10 cells (5 × 10^5^) and PKH26-stained platelets (9 × 10^8^) were injected into opposite tail veins of *Cx3cr1^gfp/+^* mice. After 8 or 24 hours, isolated lungs were placed in a specially designed chamber with a coverslip glass (0.16–0.19 mm thick) at its bottom. To visualize lung endothelium, anti-CD31–PE antibody (50 mg/kg; 102408, BioLegend) was injected in the vena cava 5 minutes before sacrifice, followed by vena cava ligation. Lungs were inflated with 0.5 ml of air and remained inflated during the imaging (27, 31, 81). Tumor cell extravasation was evaluated visually from microscopic FOV or through reconstruction of tumor cells and vessel surface with Imaris software (versions 8.2 and 9, Bitplane). The size of platelet microclots and monocyte/macrophage clusters was calculated through MATLAB (R2017a) code written in-house.

### Spontaneous metastasis assay.

5 × 10^5^ 4T1-GFP cells were injected s.c. in the right flank of female BALB/c mice. Tumors were measured using a digital caliper, and the volume was calculated as height × length × width × π/6. When the tumor reached 20–30 mm^3^, mice were randomly allocated to 4 treatment groups (vehicle, aspirin, SC-560, or picotamide). When tumor reached 800 mm^3^, lungs were perfused/isolated for ex vivo imaging of the left lung. Lung and liver metastatic nodules were counted.

### Isolation of CTCs and Transwell invasion assay.

Blood from BALB/c mice was drawn in syringes containing ACD buffer (1:5 vol/vol). Whole blood was diluted in an equal volume of PBS, layered on Ficoll-Paque PLUS media solution (GE Healthcare), and centrifuged at 400 *g* for 30 minutes at 19°C, according to the manufacturer’s instructions. The mononuclear cell layer was isolated, and GFP^+^ cells were counted as CTCs.

For Transwell migration and invasion assays, 5000 CTCs were resuspended in serum-free DMEM and seeded on a Transwell insert (8 μm pore size; BD Biosciences) coated with growth factor reduced Matrigel matrix (2 μg/μl; BD Biosciences). The bottom chamber contained DMEM supplemented with 2% FBS as chemoattractant. After 20 hours, cells adherent to the bottom well were fixed with 2% PFA, and GFP^+^ cells were counted with a Celigo S Imaging Cytometer (Nexcelom Bioscience LLC).

### Premetastatic niche formation assay.

5 × 10^3^ B16F10 cells were injected s.c. in the back of female C57BL/6 or *Cx3cr1^gfp/+^* mice. Two days after the tumor became palpable (12–15 days from injection), mice were treated with aspirin (medium dose) for 5 days. Treatment was interrupted 2 days before the i.v. injection of 2.5 × 10^5^ B16F10 cells. Lungs were collected either just before (*Cx3cr1^gfp/+^* mice, recruitment of myeloid cells) or 2 weeks after tumor cell injection (C57BL/6, metastatic lung nodules).

### Isolation and culture of LMVECs.

Primary LMVECs were isolated as previously described (76). Briefly, lungs from female C57BL/6 mice were dissected, digested in a 0.5-mg/ml collagenase solution for 1 hour at 37°C, and filtered through a 70-μm cell strainer (Thermo Fisher Scientific). Suspended cells were washed, blocked with 10 μg/ml murine IgG (I5381, Sigma-Aldrich), and stained with isolectin-B4–FITC (L2895, Sigma-Aldrich), anti-CD31–PE (102408, BioLegend), and anti-CD105–APC (120414, BioLegend) antibodies, diluted to 1 μg/ml in PBS with 2.5% FBS. Isolectin-B4^+^CD31^+^CD105^+^ cells were sorted using a Beckman Coulter Legacy MoFlo MLS High Speed Cell Sorter and cultured.

### Measurement of prostanoids.

After 2 days of treatment with vehicle or drugs, blood was collected through the vena cava of terminally anesthetized C57BL/6 mice. For serum, blood was left to clot for 30 minutes at room temperature and centrifuged at 850 *g* for 15 minutes at 4°C. For plasma, blood collected with ACD buffer (1:10 vol/vol) was centrifuged at 1000 *g* for 15 minutes at 4°C. B16F10 cells (3 × 10^4^) and/or platelets (50 × 10^6^) were coincubated for 24 hours, followed by centrifugation of conditioned medium at 1300 *g* for 10 minutes to remove detached cells and platelets. TXB_2_ concentration was measured through a Thromboxane B_2_ EIA kit (Cayman Chemical). 6-Keto-PGF_1α_ concentration was measured in anticoagulated plasma through a 6-keto-PGF_1α_ ELISA kit (Enzo Life Sciences).

For ex vivo PGE_2_, whole anticoagulated blood was incubated with 10 μg/ml LPS (Sigma-Aldrich) or saline for 24 hours at 37°C. Plasma was assayed with a PGE_2_ ELISA kit (Abcam). For in vivo PGE_2_, C57BL/6 mice were injected with 5 mg/kg LPS or saline, and anticoagulated blood was collected through vena cava 4 hours after injection. Plasma PGE_2_ was assayed through a PGE_2_ Metabolite EIA kit (PGE_2_M, Cayman Chemical), which measures 13,14-dihydro-15-keto-PGA_2_ and 13,14-dihydro-15-keto-PGE_2_ metabolites.

### Measurement of urinary salicyluric acid.

Urine was collected from restrained C57BL/6 mice 2 weeks after i.v. injection of B16F10 and supplemented with indomethacin (10 μg/ml in DMSO; Sigma-Aldrich). Urine was centrifuged at 10,000 *g* for 15 minutes at 4°C. Five microliters of urine was mixed with 50 μl 6-methoxysalicylate (internal standard, 10 μM) and 1 ml formic acid (10 mM). To measure salicyluric acid (SUA), a 5-μl sample was injected onto the HPLC (Waters 2695) equipped with a Micromass Quattro Micro Mass spectrometer (Waters). Separation was achieved using a Kinetex XB (2.6 μm, 2.1 × 50 mm) column maintained at 35°C with eluent A (10 mM formic acid) and eluent B (acetonitrile), using a flow rate of 0.25 ml/min and a gradient of 8%–50% B over 4 minutes. SUA was detected using electrospray in negative mode with tandem mass spectrometry with a capillary voltage of 1.2 V at 194–150 (cone voltage 20 V) and internal standard at 166.9–123 (cone voltage 20 V). Concentrations were calibrated against SUA [*N*-(2-hydroxybenzoyl)glycine, Apollo Scientific].

### Tumor cell adhesion assay under flow.

A flow-based adhesion assay coupled to live cell imaging was used, as described previously (82). 1.5 × 10^4^ LMVECs were seeded in μ-Slide IV^0.4^ (Ibidi) and grown to confluence. LMVECs, platelets, and tumor cells were pretreated with vehicle, aspirin (medium dose), SC-560, or NS-398 for 2 hours (LMVECs and B16F10 cells) or 30 minutes (platelets) and washed twice. 3 × 10^5^ CMFDA-labeled B16F10 and 60 × 10^6^ PKH26-labeled platelets (resuspended in PPP) were introduced to flow on an LMVEC monolayer at 0.05 dyn/cm^2^ for 10 minutes, in the presence or absence of drugs. Shear stress was subsequently increased to 1 dyn/cm^2^ for 2 minutes to remove nonadhered cells or platelets. Image acquisition was performed using a Nikon Eclipse TE2000-E microscope (Nikon Plan Fluor 10×/0.30 Ph1 DL objective) equipped with a Hamamatsu ORCA-ER digital camera (12 frames per minute). Shear-resistant adhesion and association with platelets were quantified from 10 FOVs acquired before and after the increase of shear stress with Imaris software.

### Immunofluorescence.

Lungs were perfused with ice-cold PFA 4% and stored in sucrose 25% (Sigma-Aldrich) at 4°C for 2 days. Eighteen-micrometer sections (cryostat microtome, Bright) from snap-frozen lungs were stained for E-selectin (MA1-06506, Thermo Fisher Scientific), VCAM-1 (CBL1300, Millipore), and vWF (ab11713, Abcam) using a TSA biotin amplification system (PerkinElmer).

### Confocal microscopy.

*Z*-stack images were acquired with an inverted confocal microscope (LSM-710 and LSM-880, Zeiss) equipped with a Plan-Apochromat 20×/0.8 M27 objective. DAPI/CMAC (excitation, 405 nm; emission, 410–513 nm), GFP/Alexa Fluor 488/CMFDA (excitation, 488 nm; emission, 490–653 nm), PE/CMRA (excitation, 543 nm; emission, 548–692 nm), PKH26 (excitation, 561 nm; emission, 568–735 nm), and Alexa Fluor 633 (excitation, 633 nm; emission, 638–747 nm) were detected via a photomultiplier tube array (DAPI, CMAC, 4T1-GFP, Alexa Fluor 488, CMFDA, CMRA, PKH26, Alexa Fluor 633) or a gallium arsenide phosphide (GaAsP) array (PE, Cx_3_CR1-GFP). Channels were acquired sequentially to minimize bleed-through of emitted light. Stacks of 15–40 slices at 1- to 2-μm intervals from random FOV or tile scans of whole left lung (×10 or ×20) were acquired.

### Statistics.

Statistical analysis was performed with GraphPad Prism (version 5.02). D’Agostino and Pearson omnibus normality test was applied to assess data distribution. For normally distributed data, unpaired *t* test (2-tailed) or 1-way ANOVA with Tukey’s test or Pearson’s test was used. For non-normally distributed data, Mann-Whitney test, Kruskal-Wallis with Dunn’s multiple-comparisons post hoc test, or Spearman’s test was used. Outliers were identified through Grubbs’s test (α = 0.05, GraphPad QuickCalc outlier calculator) and excluded. Differences were considered significant with a *P* value lower than 0.05.

### Study approval.

Animal procedures were in accordance with UK Animal law (Scientific Procedures Act 1986), including local ethics approval at the University of Oxford under project license 30/3413.

## Author contributions

This project was conceived by SL, AMGB, and RJM with the methodology developed by SL, CC, MS, AMGB, ALG, PDA, SS, TDW, AJR, and RJM. The investigations were performed by SL, CC, MS, AMGB, ALG, BM, and KW. Software was developed by PDA and resources provided by PDA, SS, JAW, TDW, AJR, and RJM. Supervision of the project was provided by AMGB, TDW, AJR, and RJM. The original draft was written by SL and RJM

## Supplementary Material

Supplemental data

Supplemental Video 1

## Version 1 03/25/2019

Electronic publication

## Version 2 05/01/2019

Print issue publication
