# Supplementary material for: Aspirin blocks formation of metastatic intravascular niches by inhibiting platelet-derived COX-1/thromboxane A2
Source: J Clin Invest. 2019 Mar 25;129(5):1845–62. doi: 10.1172/JCI121985 (PMC6486338; doi:10.1172/JCI121985)
Supplement: Supplemental data [file jci-129-121985-s042.pdf]

Supplementary figures

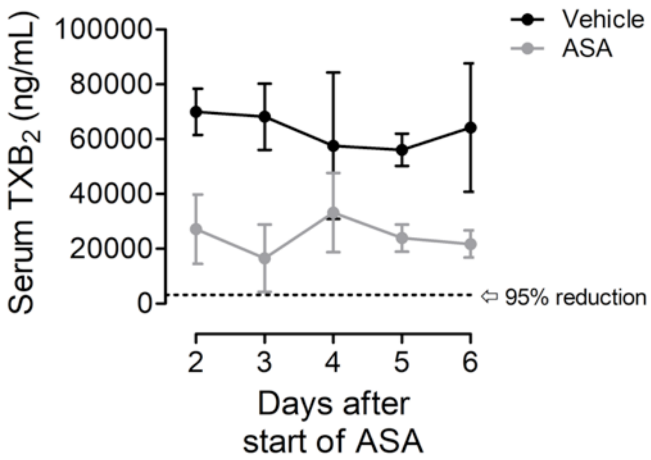

Figure S1. Low dose aspirin does not accumulate over time. Concentration of TXB_2 in serum from C57BL/6 mice treated with vehicle or aspirin (Low dose) for up to six days, with daily withdrawal of blood (*n*=4).

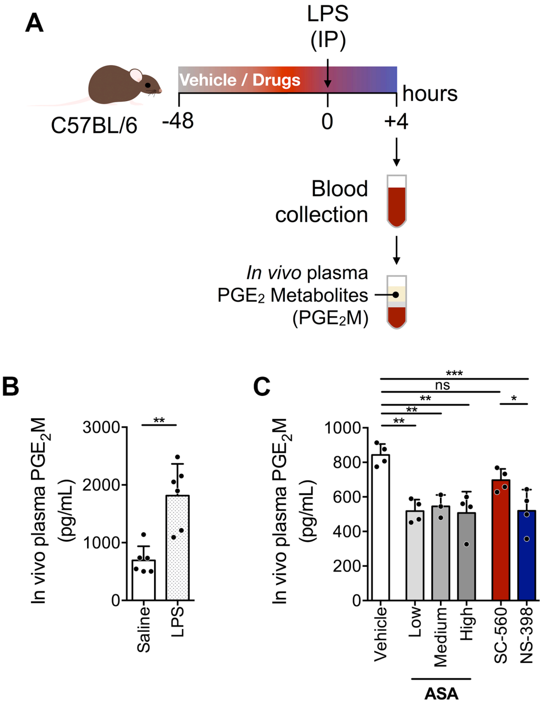

Figure S2. Aspirin and NS-398 inhibit COX-2 activity in vivo. (**A**) Experimental design for the in vivo measurement of plasma PGE_2. Mice were treated with vehicle or aspirin. After two days mice were injected IP with saline or LPS and blood was collected 4 hours later for the measurement of plasma PGE_2 Metabolites (PGE_2M, equivalent to 13,14-dihydro-15-keto PGA_2 and 13,14-dihydro-15-keto PGE_2). PGE_2M is representative of systemic COX-2 activity. (**B**) Concentration of PGE_2M in plasma from saline- or LPS-injected mice (*n*=6). (**C**) Concentration of PGE_2M in plasma derived from LPS-injected mice treated with vehicle, aspirin, SC-560 or NS-398 (*n*=4).

Data are represented as mean + SD. Unpaired t test, two-tailed (**B**); 1-way ANOVA with Tukey's Multiple Comparison test (**C**).

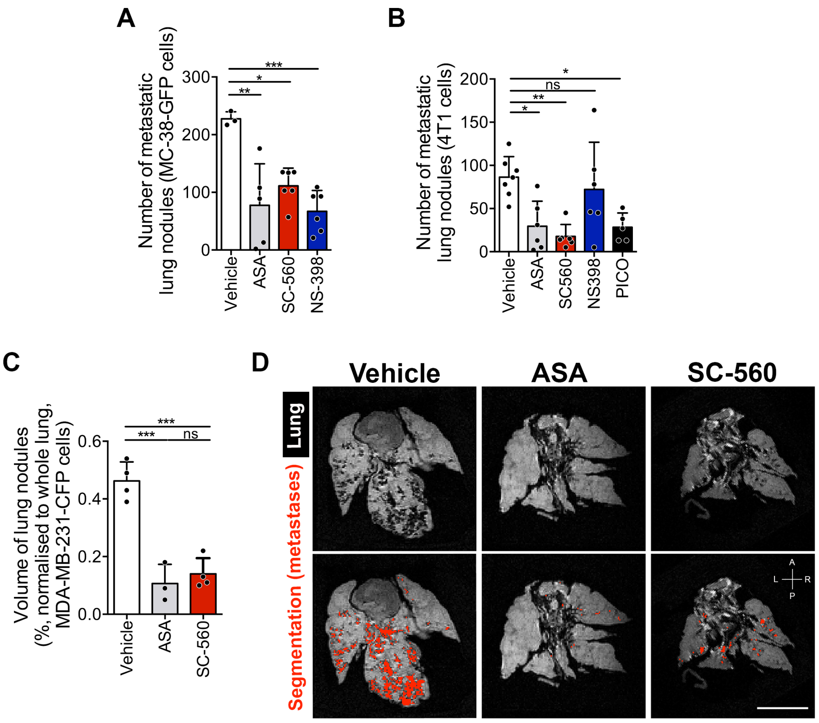

Figure S3. Aspirin reduces experimental metastasis from multiple cancer types. (**A**) Number of MC-38-GFP metastatic lung nodules in C57BL/6 mice treated with vehicle, aspirin, SC-560 or NS-398 (*n*=3, 5, 6 and 6). (**B**) Number of 4T1 metastatic lung nodules BALB/c mice treated with vehicle, aspirin, SC-560, NS-398 or PICO (*n*=7, 6, 6, 6 and 5). (**C-D**) Volume of MDA-MB-231-CFP lung nodules, normalized to total lung volume (**C**), and representative images of lungs scans (**D**) acquired by MRI on ex vivo lungs from SCID mice treated with vehicle, aspirin or SC-560 (*n*=4, 3 and 4). Segmentation of the metastasis volume in the single pane (bottom row) is represented in red. Scale bar: 5 mm.

Data are represented as mean + SD. 1-way ANOVA with Tukey's Multiple Comparison test.

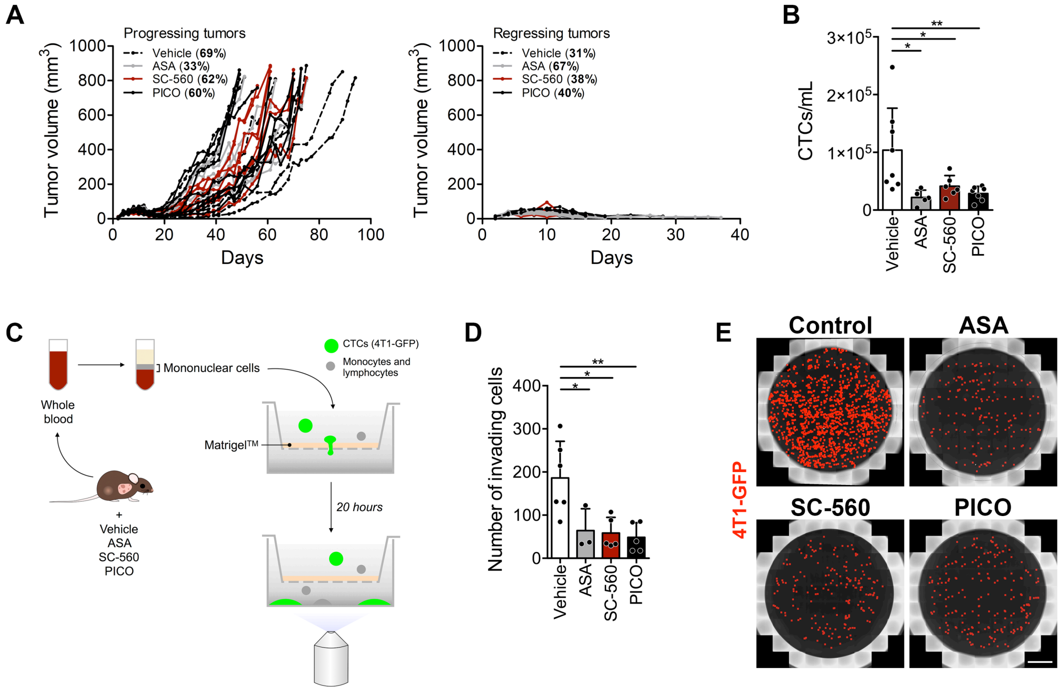

Figure S4. COX-1/TXA$_2$ inhibition reduces the number and invasiveness of CTCs. (**A**) Growth curves and incidence of progressing tumors (left panel) and regressing tumors (right panel) in BALB/c mice inoculated with 4T1-GFP cells and randomized to receive vehicle, aspirin, SC-560 or picotamide treatment (*n*=8, 5, 8 and 9 in left panel; *n*=4, 10, 5 and 6 in right panel), as in Figure 2E. (**B**) Number of 4T1-GFP CTCs in blood from BALB/c mice under vehicle, aspirin, SC-560 or picotamide treatment (*n*=8, 5, 6 and 7). (**C**) Experimental design of the CTCs transwell invasion assay. 5000 CTCs isolated from blood from 4T1-GFP tumor-bearing mice were seeded in the top chamber and counted in the bottom chamber after 20 hour of incubation. DMEM supplemented with 2% FBS was used as chemoattractant in the bottom chamber. (**D-E**) Number of invaded 4T1-GFP CTCs (**D**) counted in the bottom well of the invasion chamber (**E**). CTCs were isolated from blood of 4T1-GFP-tumour bearing BALB/c mice treated with vehicle, aspirin, SC-560 or picotamide (*n*=6, 3, 5 and 5). Scale bar: 3 mm.

Data are represented as mean + SD (**B** and **D**). 1-way ANOVA with Tukey's Multiple Comparison test.

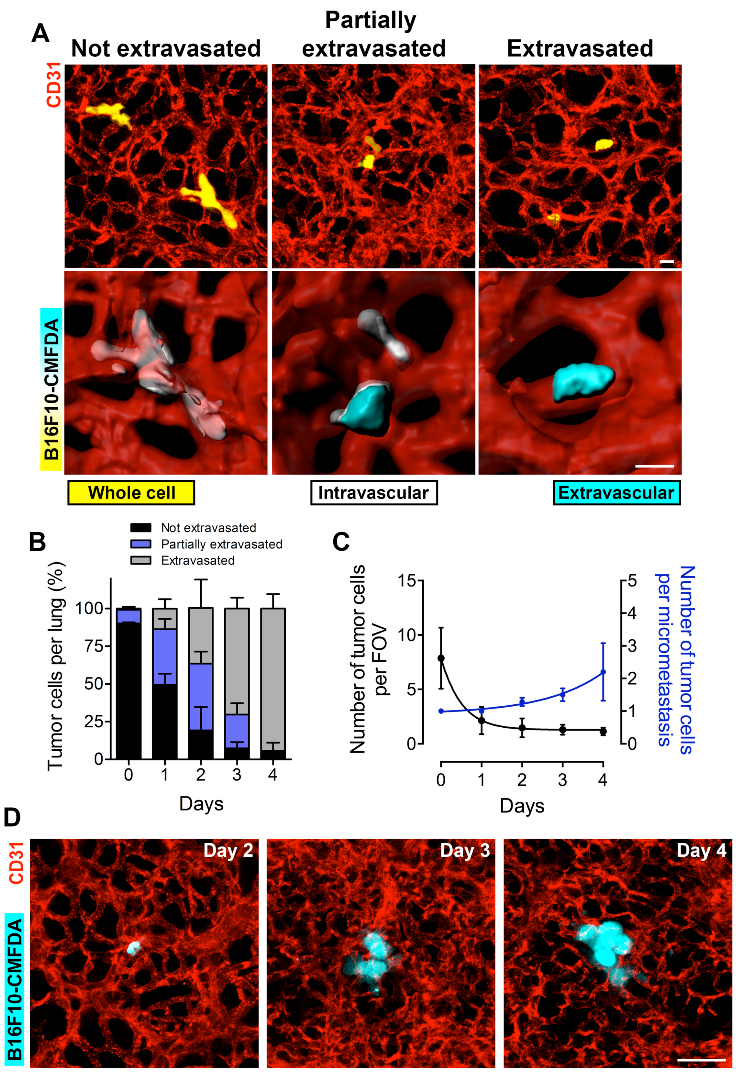

Figure S5. Kinetics of B16F10 cells extravasation in lungs. (**A**) MIP of 3D confocal stacks (top) and 3D surface reconstruction (bottom) of tumor cells (B16F10-CMFDA, yellow) in whole lungs of C57BL/6 mice intravenously injected with CD31-APC antibody to label endothelial cells (red). Tumor cells were scored as Not extravasated (left panels), Partially extravasated (middle panels) or Extravasated (right panels) according to their localization relatively to the labeled vasculature. The intravascular (white) and extravascular (cyan) portions of tumor cells are indicated in the bottom panels. Scale bars: 10 µm. (**B**) Quantification of the number of Not extravasated, Partially extravasated and Extravasated B16F10-CMFDA cells, imaged in whole lungs up to 4 days after intravenous injection (*n*=3, ≥6 tumor cells per lung). (**C**) Number of B16F10-CMFDA cells (black curve) and number of adjacent tumor cells per micrometastasis (blue line), counted in microscopic FOVs from whole lungs of C57BL/6 mice, up to 4 days after intravenous injection of tumor cells (*n*=3). (**D**) MIP of 3D confocal stacks of B16F10-CMFDA cells at different stages of proliferation, imaged in whole lungs of C57BL/6 mice. Scale bar: 30 µm.

Data are represented as mean +/± SD.

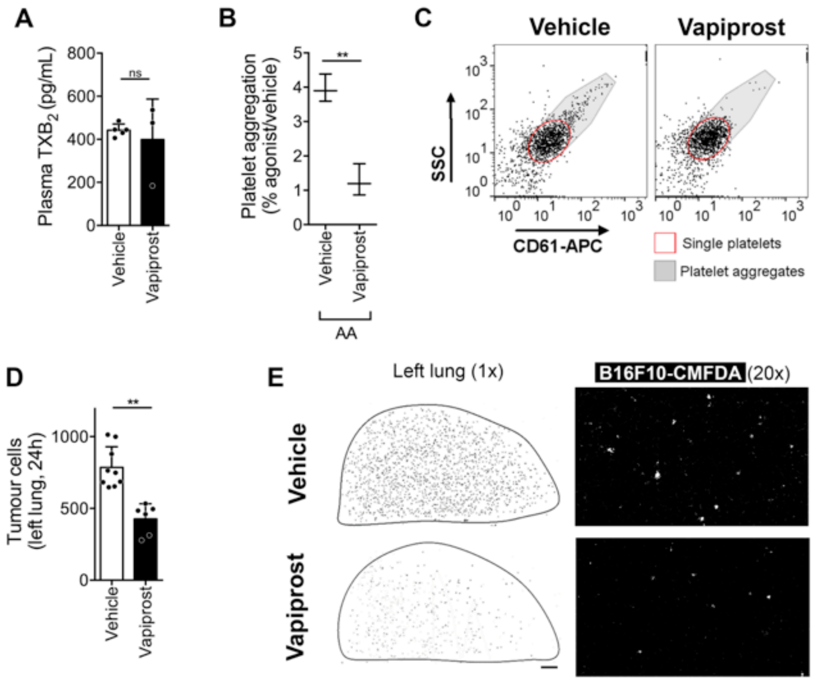

Figure S6. TP antagonism is sufficient to reduce experimental metastasis. (**A**) Concentration of TXB_2 in plasma from C57BL/6 mice treated with vehicle or vapiprost for two days (*n*=5 and 3). (**B-C**) Arachidonic acid-induced aggregation of platelets from C57BL/6 mice treated with vehicle or vapiprost (*n*=4). (**D-E**) Number of B16F10-CMFDA cells (white) (**D**) and representative tile scans (**E**) of the left lung of C57BL/6 mice treated with vehicle or vapiprost (*n*=9 and 6) 24 hours after the injection of tumor cells. Scale bars: 1 mm (black bar); 100 µm (white bar).

Data are represented as mean + SD (**A** and **D**), median ± range (**B**). Unpaired t test, two-tailed.

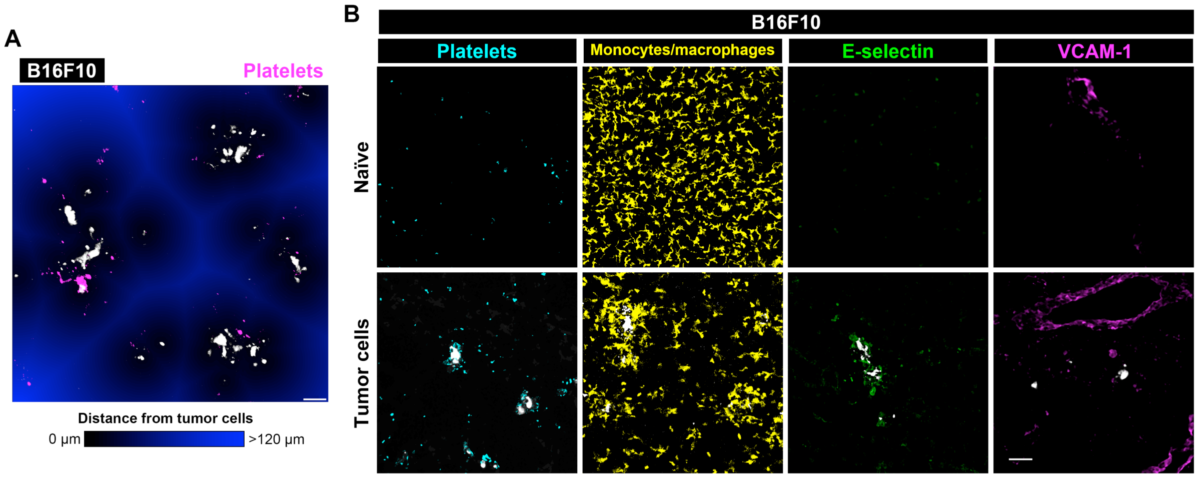

Figure S7. Tumor cell-platelet microemboli drive the establishment of the intravascular metastatic niche. (**A**) MIP of 3D confocal stack of tumor cells (B16F10, white), platelets (Plts-PKH26, magenta) and distance transformation from tumor cell surface (black to blue). Scale bar: 30 µm. (**B**) MIP of 3D confocal stacks (20x) of tumor cells (B16F10, white), platelets (Plts-PKH26, cyan), monocytes/macrophages (GFP, yellow) and endothelial activation markers (E-selectin, green, and VCAM-1, magenta; median filter) in lung sections or whole lungs from C57BL/6 mice or $Cx_3CR1^{gfp/+}$ mice not injected (Naïve, top row) or injected (bottom row) with tumor cells. Scale bar: 50 µm.

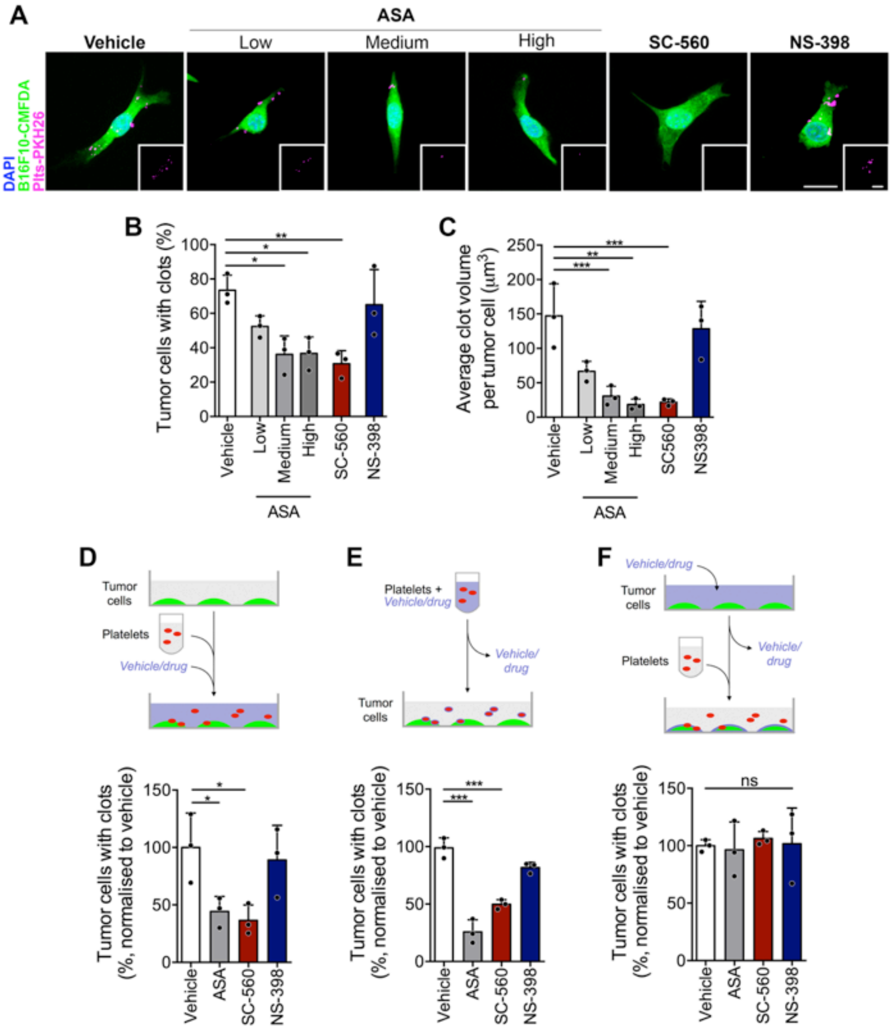

Figure S8. The inhibition of COX-1 in platelets reduces the aggregation of platelets on tumor cells in vitro. (**A**) MIP of 3D confocal stacks of tumor cells (B16F10-CMFDA, green) seeded on culture slides and incubated with platelets (Plts-PKH26, magenta) in the presence of vehicle, aspirin (Low = 0.075 mg/mL, Medium = 0.217 mg/mL and High = 1.171 mg/mL), SC-560 (60 µg/mL) or NS-398 (30.4 µg/mL). The nuclei of tumor cells were stained with DAPI after fixation (blue). Doses of drugs correspond to the expected plasmatic concentration in mice. Scale bar: 30 µm. (**B-C**) Percentage of tumor cells associated with platelets (**B**) and average volume of platelet clots per tumor cell (**C**) upon vehicle, aspirin, SC-560 or NS-398 treatment in vitro (*n*=3). (**D-F**) Percentage of tumor cells associated with platelets after treatment with vehicle or drugs of the co-culture (**D**), of platelets alone (**E**) or of tumor cells alone (**F**), quantified from confocal microscopy images (20x) (*n*=3).

Data are represented as mean + SD. 1-way ANOVA with Tukey's Multiple Comparison test.

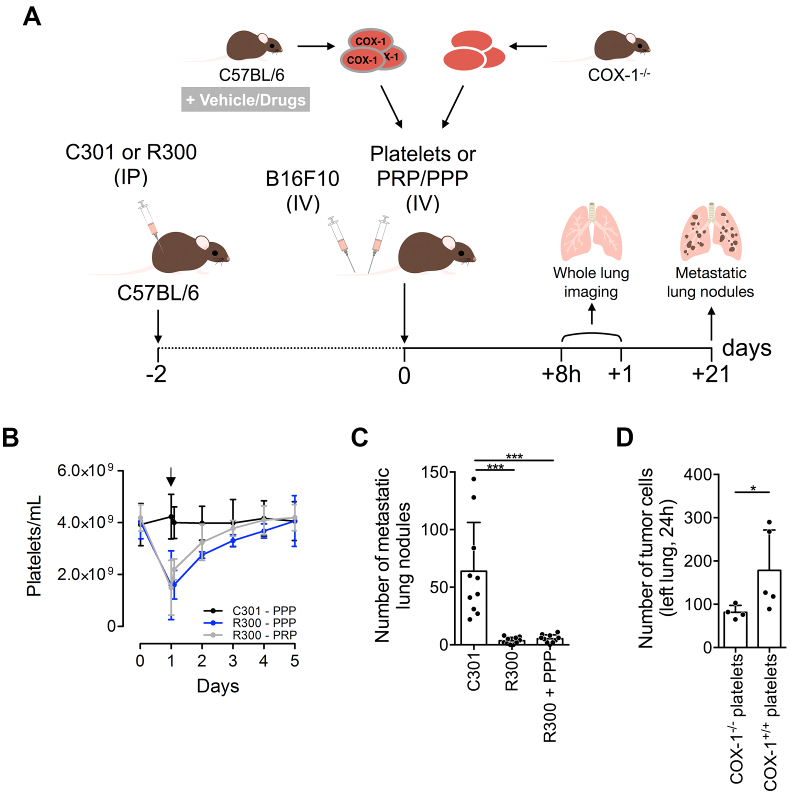

Figure S9. Platelet depletion abrogates experimental metastasis. (**A**) Experimental design for the depletion-infusion of platelets. C57BL/6 or *Cx₃cr1*$^{gfp/+}$ mice received isotype control (C301) or platelet-depleting antibody (R300). After 24 hours, B16F10 tumor cells, PPP, PRP or platelets were introduced intravenously. PRP/PPP and platelets were isolated from COX-1$^{-/-}$ mice or COX-1$^{+/+}$ mice treated with vehicle, aspirin, SC-560, NS-398 or picotamide for two days before blood withdrawal. Lungs were harvested for whole lung imaging (8 and 24 hours) or counting of metastatic lung nodules (21 days). (**B**) Platelets count in whole anticoagulated blood from C57BL/6 mice injected with C301 or R300 (day 0) and re-infused with PPP or PRP (day 1, arrow). (**C**) Number of metastatic lung nodules in C57BL/6 mice that received C301 or R300 antibody 24 hours before injection in the opposite tail vein of B16F10 cells together with or without PPP from COX-1$^{+/+}$ mice (n=10). (**D**) Total number of tumor cells in the left lung of platelet-depleted COX-1$^{-/-}$ recipient mice (n=4 and 5) at one day after intravenous injection of platelets (from COX-1$^{-/-}$ or COX-1$^{+/+}$ donor mice) and tumor cell (B16F10-CMFDA).

Data are represented as mean +/± SD. 1-way ANOVA with Tukey’s Multiple Comparison test.

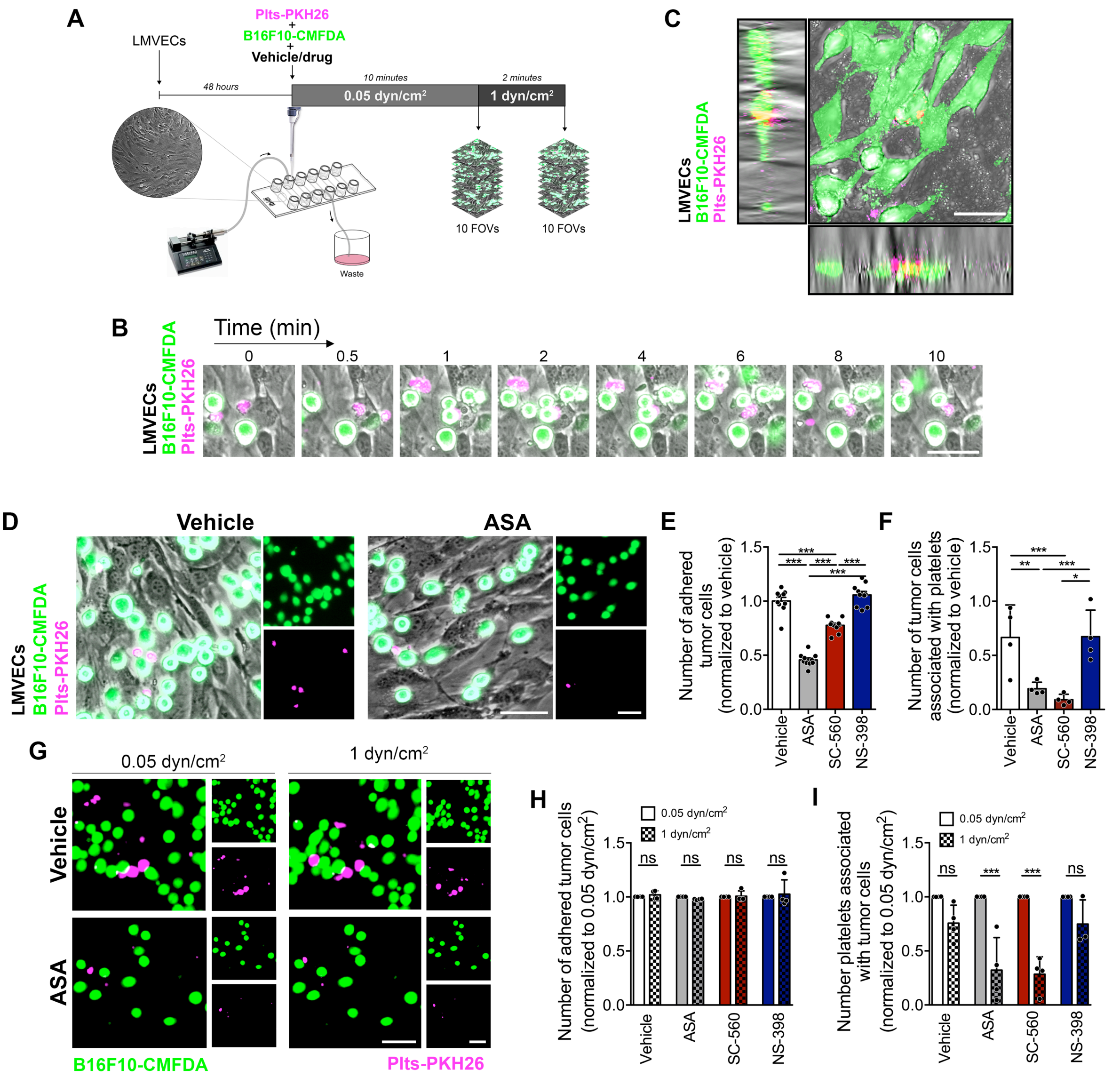

Figure S10. COX-1 inhibition reduces the adhesion of tumor cells to the lung endothelium and the shear-resistance of platelet-tumor cell interaction. (**A**) Experimental design of the tissue culture fluid flow system employed to assess the adhesion of tumor cells (B16F10-CMFDA) to LMVECs in the presence of platelets (Plts-PKH26). Vehicle or drugs were added shortly before the application of shear stress. (**B**) Selected still images of time-lapse microscopy analysis of tumor cells (B16F10-CMFDA, green) adhering to LMVECs (DIC) in the presence of platelets (Plts-PKH26, magenta) under flow at 0.05 dyn/cm^2 pressure. Scale bar: 20 µm. (**C**) MIP of 3D confocal stacks and orthogonal views of tumor cells adhering to LMVECs monolayer in the presence of platelets. Scale bar: 50 µm. (**D-F**) Selected still images (**D**), relative number of adhered tumor cells (*n*=10 FOVs) (**E**) and fraction of tumor cells associated with platelets (*n*=4 FOVs) (**F**), calculated from images (10x) of time-lapse microscopy of tumor cells adhering to LMVECs in the presence of platelets. Three independent experiments were performed. Scale bars: 100 µm. (**G**) Representative epifluorescence images (10x) of tumor cells and platelets under shear stress at 0.05 dyn/cm^2 (10 minutes, left panels) or subsequent 1 dyn/cm^2 (2 minutes, right panels) on LMVECs monolayer (not shown), all treated with vehicle,

aspirin, SC-560 or NS-398. Scale bars: 50 μm. (**H**) Relative number of tumor cells adhered to endothelial cells in the presence of platelets after 1 dyn/cm^2 shear stress (normalized to 0.05 dyn/cm^2), upon vehicle, aspirin, SC-560 or NS-398 treatment (*n*=4). (**I**) Percentage of platelets associated with tumor cells after 1 dyn/cm^2 shear stress (normalized to 0.05 dyn/cm^2), upon treatment with vehicle, aspirin, SC-560 or NS-398.

Data are represented as mean + SD. 1-way ANOVA with Tukey's Multiple Comparison test.

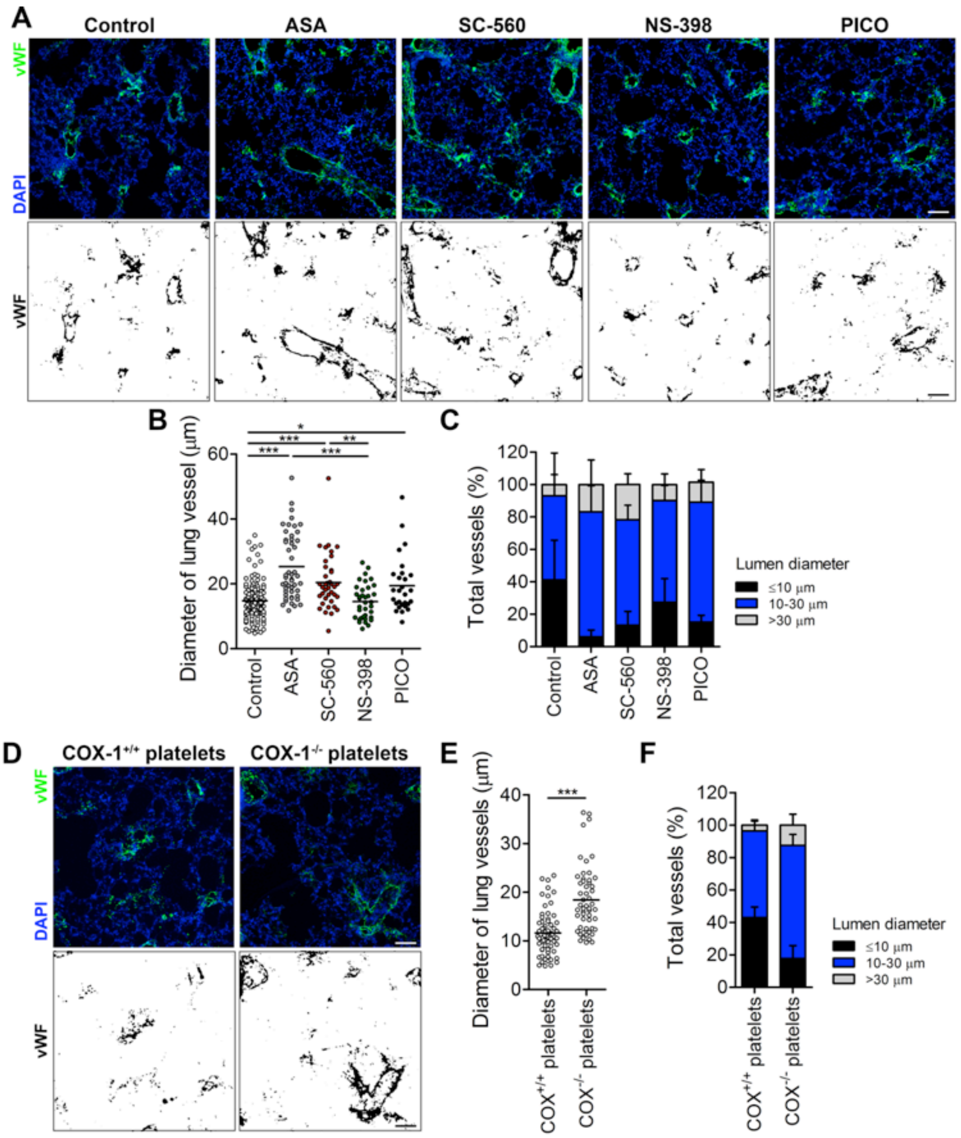

Figure S11. COX-1 inhibition is associated with reduced vasoconstriction. (**A**) MIP (median filter) of 3D confocal stacks of lung sections from *$Cx_3cr1^{gfp/+}$* mice treated with vehicle, aspirin (Medium dose), SC-560 or NS-398. Lungs were harvested at 8 hours after the injection of B16F10 cells. Endothelial cells were labeled for vWF (green) and nuclei (DAPI, blue). Bottom panels show vWF staining (inverted LUT). Scale bar: 50 µm. (**B-C**) Quantification of the luminal diameter of pulmonary vessels in lung sections from mice treated with vehicle, aspirin, SC-560 or NS-398 (*n*>25) (**B**). Three independent experiments were performed. Vessels were classified as capillaries (≤10 µm), arterioles/venules (10-30 µm) and arteries/veins (>30 µm) and normalized to the total number of vessels per FOV (*n*=3) (**C**). (**D**) MIP (median filter) of 3D confocal stacks of lung sections from C57BL/6 mice depleted of platelets and re-infused with B16F10 cells and COX-$1^{+/+}$ or COX-$1^{-/-}$ platelets. Sections were stained for vWF (green) and nuclei (DAPI, blue). Scale bar: 50 µm. (**E-F**) Luminal diameter (*n*>50; three independent experiments were performed) (**E**) and classification (*n*=3) (**F**) of pulmonary vessels in lung sections from C57BL/6 mice

depleted of platelets and re-infused with tumor cells and COX-1$^{+/+}$ or COX-1$^{-/-}$ platelets.

Data are represented as mean + dot plot (**B** and **E**) and mean + SD (**C** and **F**). 1-way ANOVA with Tukey's Multiple Comparison test (**B**); unpaired t test, two-tailed (**E**).

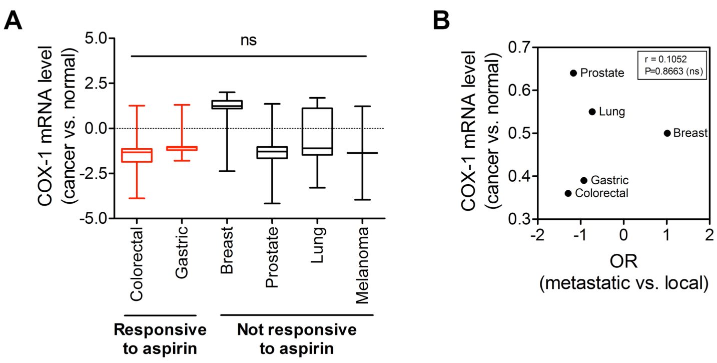

Figure S12. COX-1 expression in clinical tumor samples does not correlate with the anti-metastatic effect of aspirin (**A**) Comparative analysis of COX-1 (PTGS1) mRNA expression in human tumor specimen vs. normal tissue cohorts from ONCOMINE (Oncomine™ v4.5). Different adenocarcinomas (colorectal, gastric, breast and prostate cancer) and non-adenocarcinomas (lung and melanoma) were chosen as they represent the most frequent types of cancer reported in the metanalysis by Rothwell et al. (23). For colorectal and gastric cancer, studies involving samples of (adeno)carcinoma were selected. All cancer types express COX-1 to a similar extent to normal tissues, regardless whether aspirin reduced (colorectal and gastric, responsive to ASA) or did not reduce (breast, prostate, lung and melanoma, not responsive to ASA) the risk to become metastatic. (**B**) Correlation analysis between the relative COX-1 expression (cancer vs. normal) and the odds ratio (OR) of metastatic cancer (metastatic vs. local disease) for the most common cancer types included in the metanalysis of Rothwell et al. (23).

Data are represented as median ± IQR (**A**) or mean (**B**). 1-way ANOVA with Tukey's Multiple Comparison test (**A**); Pearson r correlation (**B**).

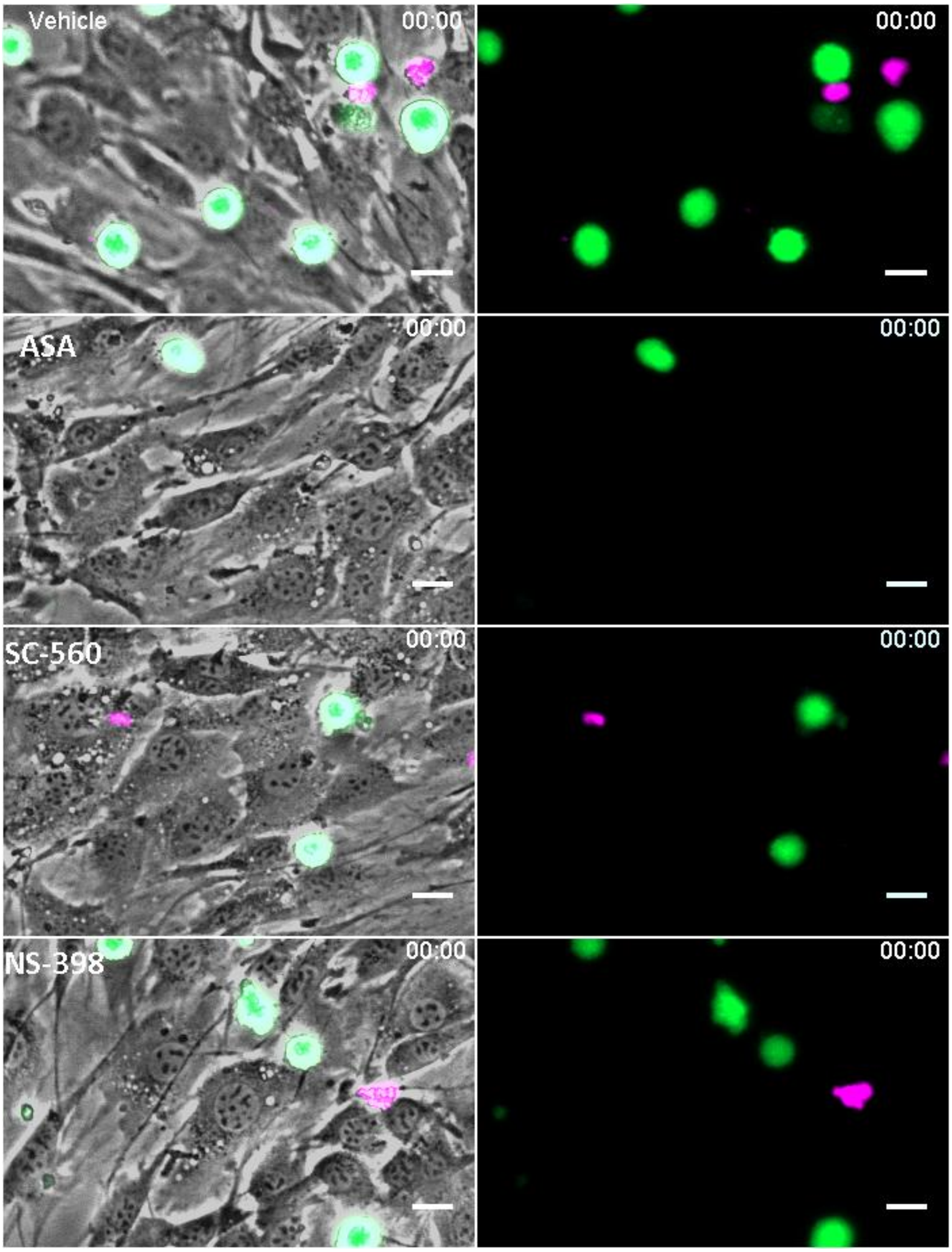

Movie S1. COX-1 inhibition reduces shear stress-resistant adhesion of B16F10 cells to LMVECs and the aggregation of platelets on tumor cells. Shear-resistant firm adhesion of platelets (PKH26-plts, magenta) and tumor cells (B16F10-CMFDA, green) to LMVECs (DIC) in presence of vehicle, aspirin, SC-560 or NS-398. Platelets and tumor cells were pushed through the channel of Ibidi µ-Slide $IV^{0.4}$ over LMVEC monolayers under low shear (0.05 dyn/cm^2). After 10 min, flow shear stress was increased (1 dyn/cm^2) to challenge non-firmly adherent platelets and tumor cells. The number of arrested tumor cells and/or platelets increased during the accumulation phase and was counted in 10 different FOV along the channel through Imaris software. Objective 10x, 12 images per min, recording time 12 min, field of view 231 µm × 152 µm of each condition. Scale bar: 30 µm.
